# Complete genome assembly and characterization of an outbreak strain of the causative agent of swine erysipelas – *Erysipelothrix rhusiopathiae* SY1027

**DOI:** 10.1186/1471-2180-14-176

**Published:** 2014-07-02

**Authors:** Amy HY Kwok, Yufeng Li, Jingwei Jiang, Ping Jiang, Frederick C Leung

**Affiliations:** 1 Bioinformatics Center, Nanjing Agricultural University, F202, South Block, Faculty of Science Complex, 1 Weigang, Nanjing 210095, China; 2 College of Veterinary Medicine, Nanjing Agricultural University, Room 4031, 4th floor, Shaw Building, 1 Weigang, Nanjing 210095, China; 3 School of Biological Sciences, University of Hong Kong, Hong Kong, SAR, China

**Keywords:** *Erysipelothrix rhusiopathiae*, Complete genome assembly, Genome characterization, Erysipelas, Virulence factors

## Abstract

**Background:**

*Erysipelothrix rhusiopathiae* is the causative agent of animal erysipelas and, to a fewer occurrences, human erysipeloid. It is ubiquitous in nature and commensal in diverse species of animals, wild or domestic, from mammals and birds to reptiles and fish. Mechanisms of its virulence and pathogenicity are poorly understood.

**Results:**

Making use of the complete genome sequencing of *E. rhusiopathiae* strain SY1027 and comparative genome analysis between the three highly pathogenic strains (SY1027, Fujisawa and ATCC19414), the genomic structure and putative functional elements, such as pathogenicity island (PAI)-like regions, potential virulence factors and horizontal transferring genes of the bacteria are identified. Strain SY1027 genome is 1,752,910 base pairs long, just 30 kilobases smaller than strain Fujisawa, with the same GC level of 36.36%. It contains 1,845 open reading frames (ORF) predicted by GLIMMER 3.02, of which 1,775 were annotated by PGAAP, 1,757 (~95.23%) were annotated by NCBI nr blast, 1,209 by COG database and 1,076 by KEGG database. 37 potential virulence factors were annotated in strain SY1027 by VFDB, while 19 (~51.35%) of them are common in the 2 strains, 7 of which are potentially related to antibiotic resistance and highly conserved (~98-100% match identity (ID)) amongst the three strains of *E. rhusiopathiae* and modestly homologous to other gastrointestinal tract-inhabiting Firmicutes (~40% match ID), e.g. *Clostridium* spp., *Enterococcus* spp. Genomic island- and pathogenicity island-like regions were also predicted, in which some showed association with tRNA and potential virulence factors.

**Conclusion:**

Complete genome sequencing of *Erysipelothrix rhusiopathiae*, the causative agent of animal erysipelas, was performed. Molecular identification of various genomic elements pave the way to the better understanding of mechanisms underlying metabolic capabilities, pathogenicity of swine erysipelas and prospective vaccine targets besides the widely used SpaA antigens.

## Background

Since its first isolation from mice in 1876, efforts have been devoted to the studies of *Erysipelothrix rhusiopathiae* (see review: [1]). *E. rhusiopathiae* is a Gram-positive bacterium that has diverse cell morphology, remarkable chemical tolerance and poorly understood cellular structure and molecular mechanisms, especially those related to its virulence in a wide range of hosts, ranging from mammals and birds to reptiles and fish. Best known as the etiological agent of swine erysipelas– a disease that can cause acute symptoms such as septicaemia or lead to chronic syndromes like arthritis and endocarditis in pigs [1], *E. rhusiopathiae* is generally believed to be transmitted via the gastrointestinal (GI) tract by intake of contaminated water or food and causes great economical loss in husbandry worldwide. Given its ubiquitous nature in the environment and reservoir of asymptomatic carriers amongst both domestic and wild animals, the prevention and control of swine erysipelas is often challenging. More than 23 serovars have so far been described [2]. However, due to the considerable variance in their morphology, host specificity and/or pathogenicity, the practicality of serotyping remains debatable. The heat labile capsule of *E. rhusiopathiae* has been associated with its resistance to phagocytosis and virulence by transposon mutagenesis study [3]. Surface proteins such as neuraminidase, SpaA antigen, two adhesive surface proteins containing the C-terminal anchoring LPXTG motif (RspA and RspB) have shown positive correlation to virulence of the bacteria [4]–[6], however, little is known about the mechanisms of its pathogenicity.

In China, outbreaks of swine erysipelas have surfaced in recent years despite its relative tranquility in the past 3 decades, with an alarming trend of developing from scattered, domestic occurrences in a few farms to systemic outbursts in provincial scale. Three representative local reports, which are only available in Chinese, are included in Additional file 1. In present study, we isolated an outbreak field strain SY1027 from SiYang, Jiangsu and completed its bacterial genome sequencing and assembly. The complete genome of *E. rhusiopathiae* strain Fujisawa has just recently been published [7]. Together with the draft genome of strain ATCC19414, data-mining of potential virulence factors, especially those related to capsular protein biosynthesis, would offer us a more reliable comparative genomic analysis and a better understanding of the common genomic structure and a more precise prediction of potential virulence factors for improvement of vaccine targets or strategy of disease control.

## Methods

### Isolation and total DNA extraction of *E. rhusiopathiae* strain SY1027

*E. rhusiopathiae* strain SY1027 was collected from a pig farm in SiYang, Jiangsu. Anticoagulant-treated blood was collected from diseased pigs by in-house veterinarians from pig farms following the Nanjing Agricultural University Animal Ethics Committee guidelines (Approval No. IACECNAU20100902) and sent to our laboratory. The sample was inoculated in Martin’s broth supplemented with 400 μg/ml neomycin and 70 μg/ml vancomycin. A single colony was isolated by inoculation of the culture on streaked agar plate supplemented with horse serum.

The single colony of the bacterium was inoculated in Modified Feist Broth (6 g glucose, 5 g proteose peptone no.2 (BD BioSciences, San Jose, US), 5 g yeast extract, 0.5 g L-arginine, 0.5 ml Tween 80 (oleic acid) in 1 L of 0.2 M sodium phosphate buffer (pH8.0) [8] supplemented with 5% (v/v) fetal bovine serum (Invitrogen, Grand Island, US) and 50 μg/ml kanamycin and grown to its exponential growth phase. Bacteria were harvested by centrifuge and its genomic DNA was extracted according to the JGI bacterial DNA isolation CTAB protocol (http://1ofdmq2n8tc36m6i46scovo2e.wpengine.netdna-cdn.com/wp-content/uploads/2014/02/JGI-Bacterial-DNA-isolation-CTAB-Protocol-2012.pdf).

### Pyrosequencing and complete genome assembly of *E. rhusiopathiae* strain SY1027

To confirm the purity of the genomic DNA of *E. rhusiopathiae* strain SY1027, 16S rDNA-specific region was amplified and 20 individual positive clones were sequenced by Genetic Analyzer 3130 (Invitrogen, Grand Island, US). BLASTn analysis [9] revealed that *E. rhusiopathiae* strain SY1027 gDNA sequences have high similarity to those from the other 2 strains of *E. rhusiopathiae* publicly accessible. The quality and quantity of genomic DNA were evaluated by 0.7% agarose gel electrophoresis and Nanodrop2000 (Thermo Scientific, Waltham, US), and using the Quant-iT Picogreen dsDNA kit (Invitrogen), respectively.

A whole genome shotgun library was generated with 500 ng of *E. rhusiopathiae* strain SY1027genomic DNA. The shotgun sequencing procedure was performed using 454 GS Junior General Library Preparation Kit, following the manufacturer’s instruction (Roche, Basel, Switzerland). In addition, an 8 kb span paired end library was generated with 15 μg of *E. rhusiopathiae* strain SY1027 genomic DNA. The paired end sequencing procedure was performed using 454 GS Junior Paired end Library Preparation Kit, following the manufacturer’s instruction (Roche). Paired end reads were used as orientation guide for assembling the contigs into scaffolds. The DNA libraries were amplified by emPCR and sequenced by FLX Titanium sequencing chemistry (Roche). One shotgun run and one paired end run were performed on individual libraries prepared with same genomic DNA sample. After sequencing, the raw data were assembled by Newbler 2.7 (Roche) with default parameters. Primer pairs were designed along the sequences flanking the gap regions for PCR gap filling. The complete genome of *E. rhusiopathiae* strain SY1027 was submitted to NCBI Genbank and is publicly accessible [GenBank: CP005079].

### Genome annotation of *E. rhusiopathiae* strain SY1027

Glimmer 3.02 [10] was used for gene prediction in *E. rhusiopathiae* strain SY1027 complete genome. All predicted ORF sequences were translated into amino acid sequences by in-house Perl scripts. BLASTp [9] was applied to align the amino acid sequences against the NCBI non-redundant (nr) database (January, 2013). Amino acid sequences with alignment length over 90% of its own length and over 40% match identity were chosen and the description of the best hit (with highest alignment length percentage and match identity) was assigned as the annotation of predicted gene. Intergenic regions were annotated by RepeatMasker (http://www.repeatmasker.org) with default parameters. For fair comparison, genome annotation was simultaneously conducted using PGAAP (NCBI).

### Functional gene analysis

BLASTp [9] was applied to align the amino acid sequences against the COG database [11]. Amino acid sequences with alignment length over 90% of its own length and over 20% match identity were chosen and the description of the best hit (with highest alignment length percentage and match identity) was assigned as the annotation of predicted gene. All annotated genes were then classified based on their COG classes and compared to that of *E. rhusiopathiae* strain Fujisawa.

### Pathway analysis

GLIMMER-predicted ORF sequences of *E. rhusiopathiae* strain SY1027were translated into amino acid sequences by in-house Perl scripts. All sequences were submitted to KEGG database [12] for automatic pathway annotation (http://www.genome.jp/kaas-bin/kaas_main) and then manually downloaded and curated by in-house Perl scripts.

### Virulence gene analysis

BLASTp [9] was applied to align the amino acid sequences against the VFDB database [13]. Amino acid sequences with alignment length over 90% of its own length and over 20% match identity were chosen and the description of the best hit (with the highest alignment length percentage and match identity) was assigned as the annotation of predicted gene.

### Genomic island (GI) and pathogenicity island (PAI) analyses

Genomic islands and Pathogenicity islands were annotated using IslandViewer (http://www.pathogenomics.sfu.ca/islandviewer/about.php) [14] and PAI Finder (https://www.gem.re.kr/paidb/pai_finder.php?m=f) on PAIDB [15] respectively.

### Drug resistant gene analysis

BLASTp [9] was applied to align the amino acid sequences against the ARDB database [16]. Amino acid sequences with alignment length over 90% of its own length and over 40% match identity were chosen and the description of the best hit (with the highest alignment length percentage and match identity) was assigned as the annotation of predicted gene. All annotated genes were designated by the antibiotics to which they render the bacteria resistant and compared to that of *E. rhusiopathiae* strains SY1027.

### Annotation of plasmid/phage/prophage-derived genes using ACLAME database

BLASTp [9] was applied to align the amino acid sequences against the ACLAME database [17]. Amino acid sequences with alignment length over 90% of its own length and over 40% match identity were chosen and the description of the best hit (with highest alignment length percentage and match identity) was assigned as the annotation of predicted gene. All annotated genes were classified according to their corresponding potential horizontal transferring vectors (“virus” or phages in bacteria, plasmid or prophage) and then compared to that identified in strain Fujisawa.

### Comparative genomic analysis of *E. rhusiopathiae* strain SY1027 and strain Fujisawa

Complete genomes of *E. rhusiopathiae* strain Fujisawa [GenBank: NC_015601] was downloaded from NCBI Genbank. Orthologous genes were identified by reciprocal BLAT [18] using GLIMMER-predicted *E. rhusiopathiae* strains SY1027 and Fujisawa genes. Predicted genes of *E. rhusiopathiae* strain SY1027 which are found as single copies and with 90% minimum alignment length in strain Fujisawa were designated as the core genes.

## Results and discussion

### Sequencing and assembly of *Erysipelothrix rhusiopathiae* strain SY1027 complete genome

*Erysipelothrix rhusiopathiae* strain SY1027 genome was sequenced and its complete *de novo* assembly was achieved by one shotgun run and one 8 kb-span paired end run followed by PCR gap filling. A total of 65,982 raw shotgun reads (26,499,381 bases) and 65,032 raw paired end reads (19,179,868 bases) were generated, ~99.83% and ~85.72% of them, respectively, were aligned into 59 contigs and 4 scaffolds, yielding an average sequencing depth of ~23-fold. Average read lengths for the shotgun run and the paired end run are ~400 bp and ~300 bp, respectively. The largest scaffold is 1,733,324 base pairs (bp) in size and contains 43 large contigs, while that of the N50 contig is 73,030 bp, indicating that the raw assembly is highly continuous. After PCR gap filling by Sanger sequencing, the complete circular genome of *E. rhusiopathiae* strain SY1027 is 1.7 Mb (i.e. 1,752,910 bp) with 36.36% GC content, which is just ~30 kb smaller than and share the same GC content to that in strain Fujisawa (Table 1).

**Table 1:** **Summary of *E. rhusiopathiae *** **strains SY1027, Fujisawa and ATCC19414 genomes**

		***E. rhusiopathiae*** **strains**		
		**SY1027**	**Fujisawa**	**ATCC 19414**
**Total genome size (bp)**		1752910	1787941	1750000^#^
**GC level (%)**		36.36	36.36	N.A.
**Predicted ORF**		1845 (Glimmer)	1780 (Genbank)	1717 (Genbank)
**(method)**		1775 (PGAAP)		
**Annotated ORF (%^)**				
Against KEGG DB		1076 (58.32)	1073 (60.28)	1080 (62.90)
Against NCBI *nr*DB		1757 (95.23)	1697 (95.34)	1645 (95.81)
Against COG DB		1209 (65.53)	1123 (63.09)	1423 (82.88)
Against ACLAME DB		226 (12.25)	223 (12.53)	335 (19.51)
	plasmids	197 (10.68)	187 (10.51)	277 (16.13)
	phages	5 (0.27)	10 (0.56)	14 (0.82)
	prophages	24 (1.30)	26 (1.46)	44 (2.56)
Against VFDB		37 (2.01)	27 (1.52)	68 (3.96)
Against ARDB		7 (0.38)	7 (0.39)	14 (0.82)
Against PAIDB		5 (0.27)	9 (0.51)	N.A.
Repetitive seq in bp (%*)		9393 (0.54)	11225 (0.64)	N.A.
	Small RNA	5889 (0.34)	8032 (0.46)	N.A.
	Low complexity	3131 (0.18)	2874 (0.16)	N.A.
	Interspersed repeats	91 (0.01)	91 (0.01)	N.A.

Footnotes: ^#^denotes the approximate number according to public database; ^denotes number of genes in percentage to total number of predicted open-reading frames by Glimmer 3.02; *denotes length of sequence in percentage to total genome length; DB – abbreviation for database; seq– abbreviation for sequence; N.A. – non-applicable as strain ATCC19414 genome is only a draft assembly.

### Genome annotation of *E. rhusiopathiae* strain SY1027

**Figure 1:**
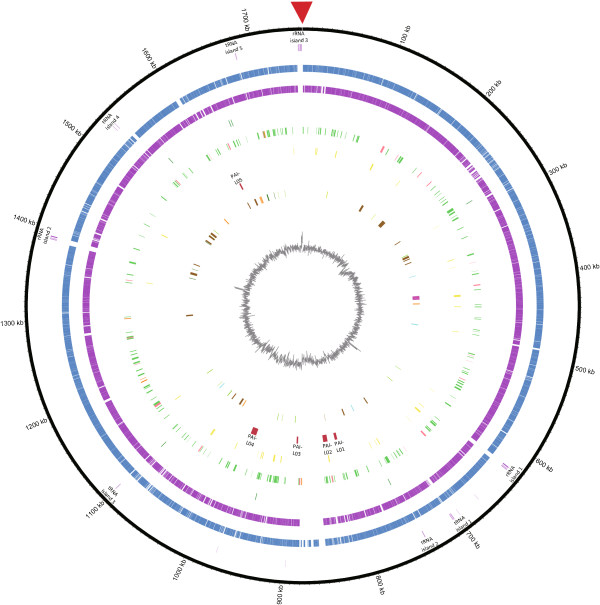
**Circular representation of *E. rhusiopathiae *strain SY1027 genome.** From the outer to inner layers, the circle shows (i) nucleotide positions in kilobases (kb) (black); (ii) RNA region whereas *rrn* operons(light purple) and tRNA islands (dark purple) are labeled accordingly; (iii) PGAAP-annotated CDSs encoded by plus strand (red) and minus strand (dark yellow); (iv) NCBI non-redundant (nr) database-annotated CDSs (blue); (v) genes shared with strain Fujisawa (dark blue); (vi) ACLAME database-annotated potential horizontal transferring genes, classified by their putative origins–plasmid (green), prophage (light red) and phage (orange); (vii) ARDB-annotated potential antibiotics resistance genes (dark red); (viii) percent G + C content (gray).

1,845 open reading frames (ORF) were predicted by GLIMMER version 3.02, and 1,757 of them (i.e. 95.32%) were annotated by BLASTp search against the NCBI non-redundant (nr) database (Mar., 2013). The full annotation result was attached as Additional file 2. Simultaneous annotation using PGAAP yielded 1,775 CDS (NCBI).Both numbers of annotated CDS were similar to that in strain Fujisawa (Table 1). 10 rRNA and 53 tRNA were identified via PGAAP. Majority of them were arranged as large RNA islands– 3 ribosomal RNA (*rrn*) operons (loci located on nucleotide positions of 623,505 to 626,780 bp, 1,386,290 to 1,391,831 bp, and 1,747,947 to 1,752,878 bp respectively) and 5 tRNA islands (located on 703,967 to 705,956 bp, 740,461 to 741,131 bp, 1,099,256 to 1,099,999 bp, 1,526,860 to 1,530,608 bp, and 1,680,522 to 1,681,399 bp respectively). The *rrn* operons are arranged in the typical order of 16S, 23S and 5S rRNA genes, with the exception of *rrn* operon 2 which is composed of an additional copy of 5S rRNA gene (data not shown). In comparison to strain Fujisawa (7 *rrn* operons), a considerably fewer number of 3 *rrn* operons are present in strain SY1027 genome (Table 1). The loci of PGAAP-annotated CDS, NCBI *nr* annotated CDS and various RNA islands were labeled in the circular representation of the SY1027 genome (Figure 1).

### Comparative genomic analysis of *E. rhusiopathiae* strain SY1027

**Figure 2:**
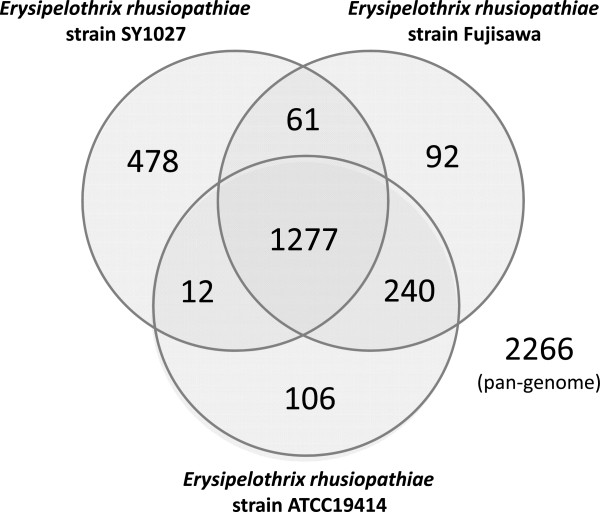
**Pan-genome between *E. rhusiopathiae *strains.** This Venn diagram is not drawn in proportion and aims only for the illustration of pan-genome and distribution of common genes including core genes. Circles represent genomes, overlapping regions between circles indicate genes shared with respective genomes. Numeral figures within respective regions denote the number of genes found therein.

**Figure 3:**
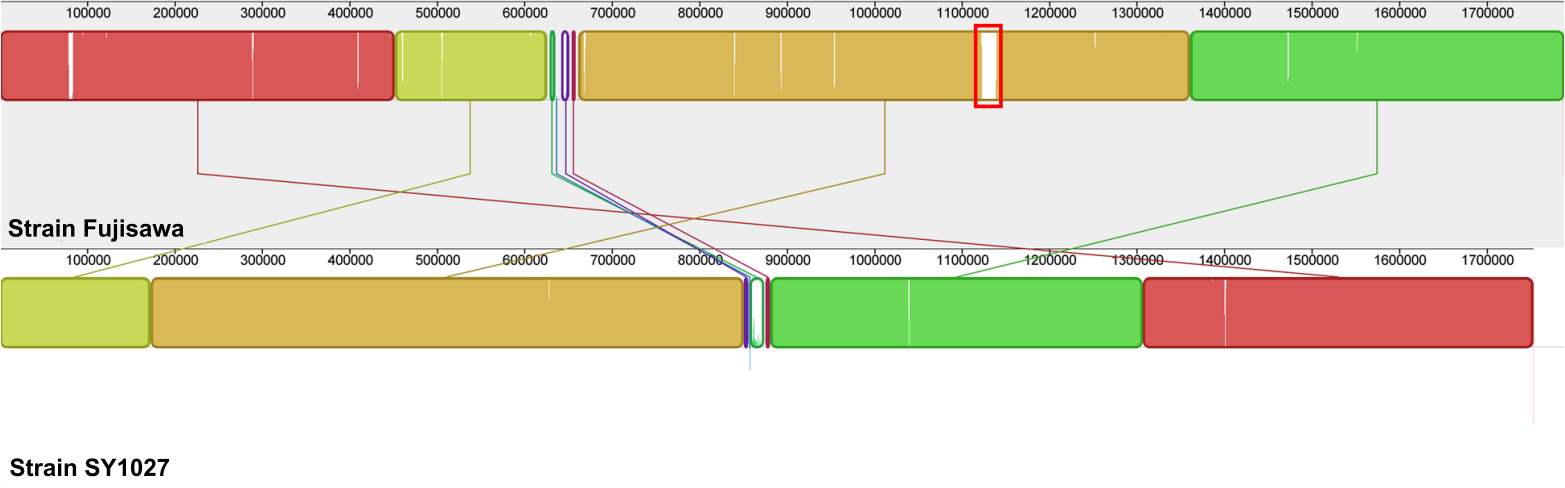
**Mauve alignment between *E. rhusiopathiae *strains SY1027 and Fujisawa genomes.** The complete genome assemblies are depicted as filled colored blocks in the order they are presented in the respective genomes. The short unmatched stretch found from ~1120 to ~1140 kb in Fujisawa genome is marked by a red box.

1,277 core genes (~56.35% of pan-genome) were identified in the pan-genome (consisted of 2,266 genes) of the three *E. rhusiopathiae* strains (Figure 2). 61 genes are shared only between strains SY1027 and Fujisawa but not ATCC19414, and only 12 between strains SY1027 and ATCC19414, whilst a far greater number of 240 genes are shared between strains Fujisawa and ATCC19414. Similarly, a greater number of 478 strain-specific genes were identified in strain SY1027, in comparison to 92 in strain Fujisawa and 106 in strain ATCC19414. These figures imply that the strains Fujisawa and ATCC19414 may be more similar to each other than to our strain SY1027, despite preliminary serotyping results that strain SY1027 is of serotype 1a, same with the other two strains (unpublished data).Synteny between the two complete genomes of strains SY1027 and Fujisawa is generally conserved, apart from a rearrangement observed at the ~850-875 kb region on strain SY1027 (which corresponded to ~625-650 kb region on strain Fujisawa) and an unmatched stretch between the two strains which is marked by a red box in Figure 3.

As suggested by a previous phylogenetic analysis after completion of strain Fujisawa genome sequencing [7], *Erysipelothrix* spp. form a distinct secluded group sandwiched between the *Firmicutes* and *Mollicutes*. Since neither draft nor complete genomes of closely related species e.g. *E. tonsillarum* and *E. inopinata* were available on GenBank, while those less closely related ones have already been explored in the previous article, hence phylogenetic tree was not constructed in our present study.

### COG annotation and comparative analysis of *E. rhusiopathiae* strain SY1027 with strains Fujisawa and ATCC19414

**Figure 4:**
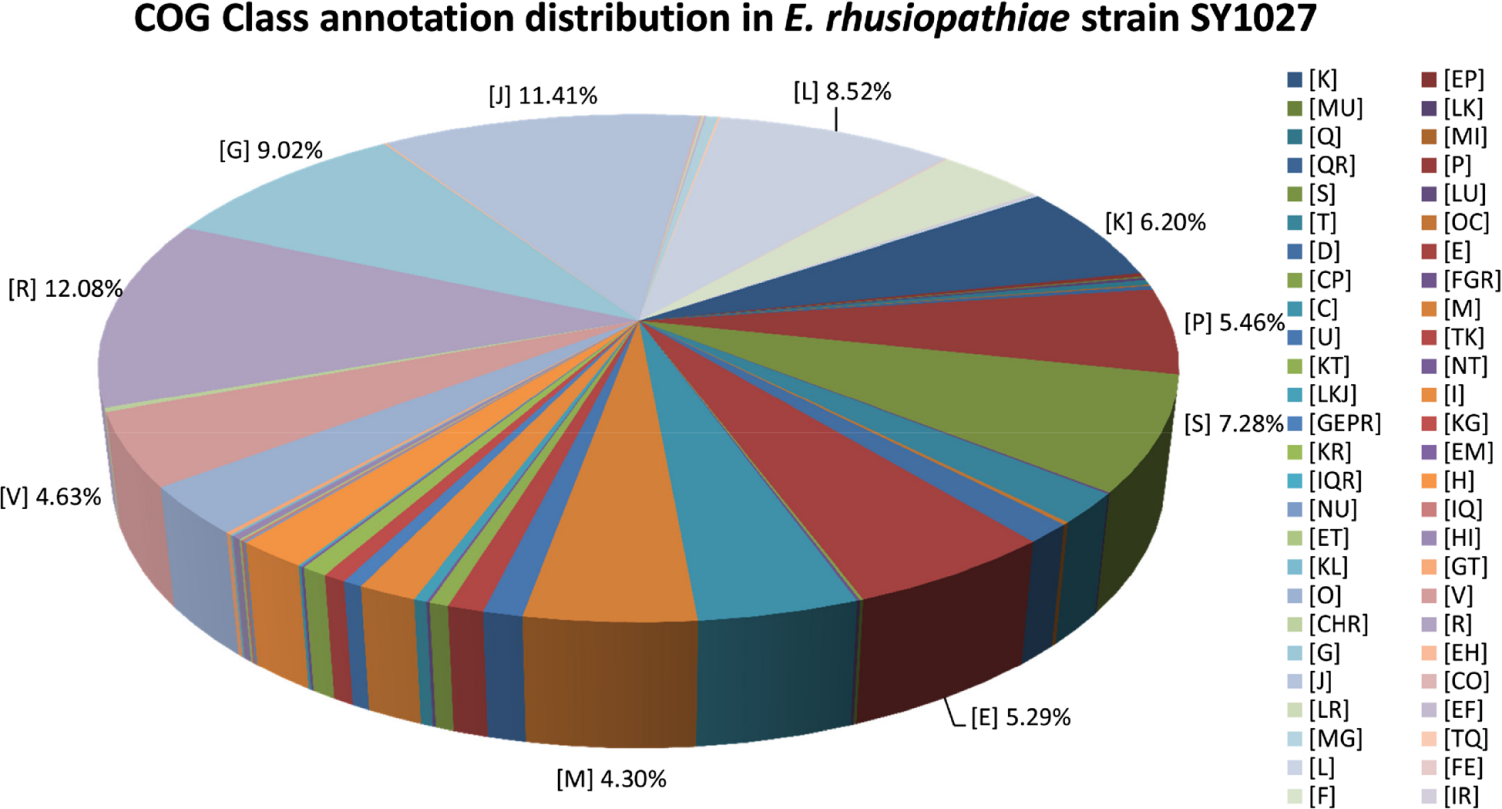
**COG class annotation distribution of *E. rhusiopathiae *strain SY1027 genome.** The COG-annotated genes are grouped under their respective COG classes. Only their class abbreviations are used in this graph, their corresponding class descriptions are listed as below: N, Cell motility; Q, Secondary metabolite biosynthesis, transport and catabolism; U, Intracellular trafficking, secretion, and vesicular transport; D, Cell cycle control, cell division, chromosome partitioning; T, Signal transduction mechanisms; I, Lipid transport and metabolism; H, Coenzyme transport and metabolism; V, Defense mechanisms; O, Posttranslational modification, protein turnover, chaperones; C, energy production and conversion; F, Nucleotide transport and metabolism; P, Inorganic ion transport and metabolism; M, Cell wall/membrane/envelope biogenesis; K, Transcription; L, Replication, recombination and repair; E, Amino acid transport and metabolism; G, Carbohydrate transport and metabolism; S, Function unknown; R, General function prediction only; J, Translation, ribosomal structure and biogenesis. Percentages of the top ten classes are labeled for easy reference.

1,209 (~65.53%) of all 1,845 predicted ORF were annotated by BLASTp search against NCBI COG database. The top ten COG classes they were annotated in were [R] General function prediction only (~12.08%), [J] Translation, ribosomal structure and biogenesis (~11.41%), [G] Carbohydrate transport and metabolism (~9.02%), [L] Replication, recombination and repair (~8.52%), [S] Function unknown (~7.28%), [K] Transcription (~6.20%), [P] Inorganic ion transport and metabolism (~5.46%), [E] Amino acid transport and metabolism (~5.29%), [V] Defense mechanisms (~4.63%), and [M] Cell wall/membrane/envelope biogenesis (~4.30%). As expected, majority of the genes were involved in basic cellular functions, such as replication, transcription, translation and metabolism, however, up to ~19.36% of them only have predicted or unknown functions on COG database. COG class distribution of strain SY1027 was illustrated (Figure 4). Numbers and percentage of each COG class were tabulated as Additional file 3 and their detailed annotations attached as Additional file 4.

Similar numbers of COG-annotated genes were found in strains Fujisawa (1,123 genes; 63.09%) and ATCC 19414 (1,423 genes; 82.88%) (Table 1). Percentage of gene annotation in ATCC 19414 is possibly higher due to incomplete sequencing coverage and assembly. COG class distributions are highly similar between strains SY1027 and Fujisawa (Additional files 5 and 6).

### Virulence gene/pathogenicity island-like gene annotation and comparative analysis of *E. rhusiopathiae* strain SY1027 and with strains Fujisawa and ATCC19414

37 potential virulence genes (~2.01% of total predicted ORF) were identified by BLASTp search against VFDB. Categorized by their respective COG classes, their accession numbers and descriptions are listed in Additional file 7. Many of these potential virulence genes are grouped under or partially involved in cell wall/membrane/envelope biogenesis (COG class [M]) or inorganic ion transport and metabolism (COG class [P]). The former mainly includes enzymes or proteins involved in capsular polysaccharide and glycoprotein biosynthesis, and the latter include transporters for iron and magnesium uptake and manganese-dependent superoxide dismutase. Metal ions are scarce and limited in biological systems and their uptake by bacteria upon invasion of various host cells have shown positive correlation to bacterial virulence [19]–[21]. However, their functions and possible relevance to pathogenicity in *E. rhusiopathiae* remain unresolved. A modestly higher number of virulence genes were identified in strain SY1027 than in strain Fujisawa in VFDB, while the number in strain ATCC19414 might be overestimated due to its draft genome nature (Table 1).

**Figure 5:**
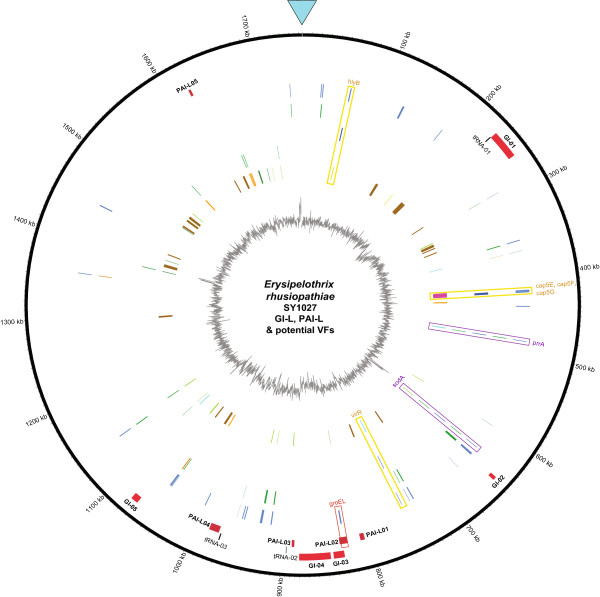
**Circular representation of GI-L, PAI-L and potential virulence factors in *E. rhusiopathiae *strain SY1027 genome.** From the outer to inner layers, the circle shows (i) nucleotide positions in kilobases (kb) (black); (ii)Island Viewer-annotated potential genomic islands (GI) are labeled accordingly (red); (iii) tRNAs flanking the potential GI or potential PAI-like regions are labeled accordingly, tRNAs found within 5000-bp flanking regions are marked in dark yellow while that found between5000 to 7000-bp flanking region is marked in grey; (iv) PAIDB-annotated PAI-like regions are labeled accordingly (dark red); (v) VFDB-annotated potential virulence factors (blue); (vi) VFDB-annotated potential virulence factors that might have originated from horizontal gene transfer are classified by their putative origins–plasmid (green) and prophage (orange);(vii) VFDB-annotated potential virulence factors that are previously mentioned in the literature are marked in dark green and their positions boxed in yellow and their gene names labeled accordingly. In cases they are predicted to be horizontally transferred from plasmids, they are boxed in purple. (viii) Other potential virulence factors suggested by previous studies that may be horizontally transferred from plasmids (green); (ix) Other potential virulence factors found in the literature suggested by previous studies, divided into 8 categories – two component system with orientation of 5’histidine kinase-3’ response regulator (cyan), two component system with orientation of 3’histidine kinase-5’ response regulator (orange), surface proteins (brown), antioxidant proteins (vivid green), phospholipases (avocado green), hemolysins (lime), proteins involved in capsular polysaccharide synthesis (pink) and others (pistachio green); (x) percent G + C content (gray). Horizontal transferring genes were annotated by ACLAME database. *groEL*, VFDB-annotated potential virulence factor that is found within putative PAI-L02, is boxed in red.

5 genomic island-like regions were annotated via Island Viewer, while 5 pathogenicity island (PAI)-like islands were identified in strain SY1027 via PAI finder in PAIDB (Figure 5, Tables 2 and 3). In comparison to the search result in VFDB, only one PAI-virulence gene (groEL) was annotated in PAI-L02, it may suggest strain SY1027 genome has PAI-like islands atypical of that found in strain Fujisawa and the present entries in the PAIDB. Though GroEL is a chaperonin widely conserved in bacteria, its association to cell adherence and virulence has been described in *Clostridium* spp. and other bacteria [22,23]. Suggestion of a PAI-island with groEL in our strain may warrant further studies in elucidating its contribution to pathogenicity, as it may offer interesting insights into potential antigenic targets for vaccine development in *E. rhusiopathiae*. In addition to the difference in GC content to the rest of the genome, tRNA were found to flank GI-01, PAI-L03 and PAI-L04, providing further support to these putative genomic islands (Tables 2 and 3). Nevertheless, the virulence of these putative regions in *E. rhusiopathiae* needs further elucidation, preferably by gene-knockout experiments. Since ATCC19414 genome was only completed to the level of scaffolds, search for GI/PAI-like islands was unfeasible.

**Table 2:** **Genomic island-like regions in *E. rhusiopathiae *** **strain SY1027 genome**

**GI #**	**Start**	**End**	**Size**	**%GC**	**flanked by tRNA**
**GI-01**	232197	263359	31162	30.5	tRNA-Leu (tRNA01)
**GI-02**	644770	648766	3996	34.6	*N.F.*
**GI-03**	831571	843384	11813	32	*N.F.*
**GI-04**	846580	880804	34224	31.3	*N.F.*
**GI-05**	1068490	1076408	7918	33.3	*N.F.*

Footnote: Genomic island-like regions were predicted by IslandPath-DIMOB on IslandViewer (http://www.pathogenomics.sfu.ca/islandviewer/about.php) [14]. *N.F*.- Not found.

**Table 3:** **Pathogenicity island-like regions in *E. rhusiopathiae *** **strain SY1027 genome**

**PAI-L #**	**Start**	**End**	**Size**	**%GC**	**flanked by tRNA**	**PAIs homologous to this region^**	No. of ORF in homo. PAI*	**Potential VF**^**ф**^
**PAI-L01**	806018	811037	5020	37.5	*N.F.*	VPI-2 (*Vibrio cholerae* O1)	8	*N.F.*
**PAI-L02**	826289	835010	8721	34.2	*N.F.*	PAI CFT073 (*E. coli* CFT073), SHI-2 (*Shigellaflexneri*M90T), HPI (*Yersinia enterocolitica*WA 314)	11	groEL
**PAI-L03**	886571	889469	2898	35.8	tRNA-Lys (tRNA02)	Not named (*Enterococcus faecalis* MMH594, V583)	7	*N.F.*
**PAI-L04**	973651	985197	11546	36.7	tRNA-Arg (tRNA03)	Not named (*Enterococcus faecalis* MMH594, V583)	15	*N.F.*
**PAI-L05**	1619897	1622633	2736	36.4	*N.F.*	Not named (*Enterococcus faecalis* MMH594, V583)	5	*N.F.*

Footnote: Pathogenicity island-like regions were predicted by PAI Finder (https://www.gem.re.kr/paidb/pai_finder.php?m=f) on PAIDB [15]. ^representative PAIs from PAIDB hits; ^Ф^as annotated by VFDB; *homo. PAI – abbreviation for homologous PAI; *N.F*.- Not found.

Apart from the public databases, a list of potential virulence factors previously suggested in *E. rhusiopathiae* were identified in the strain SY1027 genome, and they span across various categories, including surface proteins, antioxidant proteins, phospholipases, hemolysins, capsular polysaccharide biosynthesis and extracellular proteins/enzymes (Additional file 8).

Surface proteins were annotated based on their LPXTG motifs, which act as sortase recognition sequence for the covalent binding of their carboxyl termini by sortases in Gram-positive bacteria [4], or GW (glycine-tryptophan) repeats for non-covalent interactions as observed in *E. rhusiopathiae*[24]. These include SpaA antigen which is commonly used as vaccine target [5], hyaluronidases and neuraminidases which may promote bacterial-host cell surface association [25], RspA and RspB proteins which bind fibronectin and collagen I and II and may participate in biofilm formation [6]. Interestingly, a premature stop codon (TAA) was found near the middle of putative *rspA* gene (data not shown), leading to the annotation of 2 RspA-like proteins, each representing the N-terminally and C-terminally truncated proteins in strain SY1027 genome (shaded in gray in Additional file 8), instead of 1 in strain Fujisawa. Though our preliminary results suggest that these truncated proteins do not influence the serotyping of strain SY1027 (unpublished data), their conformation, collagen binding capability and influence on host-bacterial interaction may need further elucidation.

*E. rhusiopathiae* was hypothesized to have an atypical capsule that renders it escape from phagocytosis and antioxidant proteins that facilitates its survival inside polymorphonuclear leukocytes and macrophages [26]. Transposon mutagenesis study showed that the loss of a cluster of genes, putatively a polycistronic mRNA-coding operon, in strain Fujisawa created a mutant deprived of capsule and pathogenicity [3]. The operon was identified, including all 7 proteins potentially involved in capsular polysaccharide biosynthesis, in strain SY1027. Similarly, superoxide dismutase, thioredoxins, thiol peroxidase, alkyl-hydroperoxide reductases and other proteins which confer resistance to oxidative stress, were also found in strain SY1027.

Phospholipases are often considered as potential virulence factors for intracellular pathogens, hypothesized via acquisition of host membrane lipids and disruption of phagosomal membrane. In strain SY1027 genome, 6 phospholipases were identified, same as in strain Fujisawa (Additional file 8).

As *E. rhusiopathiae* is highly tolerant and almost ubiquitous in nature, two-component regulatory systems which often regulate responses to changes in the environment were also searched for in strain SY1027 genome. 9 pairs of histidine kinase-response regulator were identified– 8 shown similarity to PhoR-PhoB two-component regulatory system and the remaining one to AgrC-AgrA two-component regulatory system (Table 4).

**Table 4:** **Two-component system genes in *E. rhusiopathiae *** **strain SY1027 genome**

**Contig**_**orf**	**Gene**	**Match ID (%)**	**Order**	**Phylum**	**COG class and function annotation**
***Similar to AgrC-AgrA (exoprotein synthesis) two-component regulatory system***					
contig00001_orf00402	lin0044	39.42	Bacillales	Firmicutes	[KT]_COG3279 Response regulator of the LytR/AlgR family
contig00001_orf00401	lin0043	36.19	Bacillales	Firmicutes	[T]_COG2972 Predicted signal transduction protein with a C-terminal ATPase domain
***Similar to PhoR-PhoB (phosphate starvation response) two-component regulatory system***					
contig00001_orf00481	SP2193	43.98	Lactobacillales	Firmicutes	[TK]_COG0745 Response regulators consisting of a CheY-like receiver domain and a winged-helix DNA-binding domain
contig00001_orf00482	SP0604	27.08	Lactobacillales	Firmicutes	[T]_COG0642 Signal transduction histidine kinase
contig00001_orf00536	CAC2435	49.08	Clostridiales	Firmicutes	[TK]_COG0745 Response regulators consisting of a CheY-like receiver domain and a winged-helix DNA-binding domain
contig00001_orf00535	CAC2434	25.87	Clostridiales	Firmicutes	[T]_COG0642 Signal transduction histidine kinase
contig00001_orf00866	SP1633	52.87	Lactobacillales	Firmicutes	[TK]_COG0745 Response regulators consisting of a CheY-like receiver domain and a winged-helix DNA-binding domain
contig00001_orf00865	all3587	21.10	Nostocales	Cyanobacteria	[T]_COG0642 Signal transduction histidine kinase
contig00001_orf01180	BH0372	29.26	Bacillales	Firmicutes	[TK]_COG0745 Response regulators consisting of a CheY-like receiver domain and a winged-helix DNA-binding domain
contig00001_orf01181	BH0754	20.07	Bacillales	Firmicutes	[T]_COG0642 Signal transduction histidine kinase
contig00001_orf01262	CAC0524	41.47	Clostridiales	Firmicutes	[TK]_COG0745 Response regulators consisting of a CheY-like receiver domain and a winged-helix DNA-binding domain
contig00001_orf01261	Cgl2903	24.50	Actinomycetales	Actinobacteria	[T]_COG0642 Signal transduction histidine kinase
contig00001_orf01760	BH1153	47.39	Bacillales	Firmicutes	[TK]_COG0745 Response regulators consisting of a CheY-like receiver domain and a winged-helix DNA-binding domain
contig00001_orf01761	BS_yrkQ	27.27	Bacillales	Firmicutes	[T]_COG0642 Signal transduction histidine kinase
contig00001_orf01866^^^	CAC0564	53.33	Clostridiales	Firmicutes	[TK]_COG0745 Response regulators consisting of a CheY-like receiver domain and a winged-helix DNA-binding domain
contig00001_orf01868	BS_yvrG	24.84	Bacillales	Firmicutes	[T]_COG0642 Signal transduction histidine kinase
contig00001_orf01870^^^	CAC0830	45.02	Clostridiales	Firmicutes	[TK]_COG0745 Response regulators consisting of a CheY-like receiver domain and a winged-helix DNA-binding domain
contig00001_orf01872^^^	BS_yrkQ	25.19	Bacillales	Firmicutes	[T]_COG0642 Signal transduction histidine kinase

The highly potential virulence factors annotated both via VFDB and literature search - *hlyB, cap5E, cap5F, cap5G, prrA, sodA* and *virR* (Figure 5) – may require further elucidation on their correlation to pathogenicity, especially in intracellular survival of *E. rhusiopathiae*.

### Potential drug resistance gene in *E. rhusiopathiae* strain SY1027

7 potential drug resistance genes were annotated in *E. rhusiopathiae* strain SY1027, suggesting it may be resistant to macrolides, vancomycin and teicoplanin (Additional file 9). Their orthologs were also found in strain Fujisawa, sharing ~98-100% match ID (data not shown). Interestingly, a richer reservoir of potential drug resistance genes, both in number and variety, was annotated in strain ATCC19414 (Table 5). Our preliminary antimicrobial results suggested that despite the presence of potential macrolide-resistant genes (which share ~40-46% match IDs to ARDB entries), the antimicrobial susceptibilities to different macrolide-class drugs (e.g. erythromycin, roxithromycin and lincomycin) vary from moderate to minimal levels (unpublished data), hinting that further functional elucidation, preferably gene-knockout experiments, of the potential drug resistance genes are required to substantiate the *in silico* deduction. In addition, a previous investigation on antimicrobial susceptibilities of *E. rhusiopathiae* suggested that it has partial or complete resistance to other classes of antibiotics [27] which were not identified in our present search, hinting the presence of other potential drug resistance genes, either novel or unlisted in the ARDB, in the bacteria.

**Table 5:** **ARDB-annotated genes in *E. rhusiopathiae *** **strains SY1027, Fujisawa and ATCC19414 genomes**

	**Number of potential resistant genes to…**					
**Species**	**Macrolide**	**Vancomycin**	**Teicoplanin**	**Lincosamide**	**Tetracycline**	**Streptogramin a**
***E. rhusiopathiae*** **SY1027**	4	2	1	0	0	0
***E. rhusiopathiae*** **Fujisawa**	4	2	1	0	0	0
***E. rhusiopathiae*** **ATCC19414**	3	2	1	4	2	2

### Potential horizontal transferring elements in *E. rhusiopathiae* strain SY1027

Horizontal transferring elements are common in bacteria and generally believed to be a significant driving force in prokaryotic evolution [28]. They are mainly divided into three types based on their respective vectors – plasmids, phages (or viri) and prophages [28]. In strain SY1027 genome, 226 potential horizontal transferring elements (~12.25% of all predicted ORFs) were annotated via BLASTp against the ACLAME database (Additional file 10). Amongst them, the majority (197 genes; ~87.18%) were putatively derived from plasmids, while the rest were from phages (5 genes; ~2.21%) and from prophages (24 genes;~10.61%). Similar figures were observed in strain Fujisawa genome, with a slightly fewer number of putative plasmid-derived genes and doubled number of putative phage-derived genes. 355 potential horizontal transferring elements were found in the draft genome of strain ATCC19414, yet it might be an over-estimated figure due to incomplete or possible mis-assembly.

As observed in Figure 5, some of the potential virulence factors identified might have originated from horizontal gene transfer. Two of them –*prrA* and *sodA*–have high virulence potential owing to their identification via both VFDB and literature search. The former is the transcriptional regulator in a PrrA-PrrB two-component regulatory system related to oxygen control and intracellular replication in *Mycobacterium*[29], while the latter encodes a superoxide dismutase which may offer a mean of antioxidant defense which is critical for intracellular survival and growth in *E. rhusiopathiae*[26].

### KEGG pathway analysis of *E. rhusiopathiae* strain SY1027

KEGG pathway analysis was performed for *E. rhusiopathiae* strain SY1027, and the full list of KEGG pathway annotation was listed in Additional file 11. Strain SY1027 genome contains glucose-specific IIA component in phosphotransferase system (PTS), endoglucanase and glucose-6-phosphate isomerase, which suggest that the bacteria is capable in breaking down maltose and trehalose, cellulose and fructose, respectively. The potential of utilizing other sugars besides glucose may partly explain the ubiquitous nature of *E. rhusiopathiae* in the environment. Similar to strain Fujisawa, many critical genes in the tricarboxylic acid (TCA) cycle were found missing in strain SY1027, with the exception of fumarate hydratase, citrate CoA-transferase, dihydrolipoamidesuccinyltransferase and dihydrolipoamide dehydrogenase. Genome reduction was previously hypothesized in *E. rhusiopathiae* strain Fujisawa, given the partial loss of fatty acid biosynthesis and DNA repair system similar in the Mollicutes [7]. Likewise, strain SY1027 seems to display these characteristics. Apart from the partial loss of TCA cycle genes, only 34 genes related to DNA repair were identified.

## Conclusion

The complete genome of *Erysipelothrix rhusiopathiae* strain SY1027 was sequenced and assembled. It is 1,752,910 base pairs long, just 30 kilobases smaller than strain Fujisawa, with the same GC level of 36.36%. It contains 1845 open reading frames (ORF) predicted by GLIMMER 3.02, of which 1757 (95.23%) are annotated by NCBI nr blast, 1209 by COG database and 1076 by KEGG database. 37 potential virulence factors are annotated in strain SY1027 by VFDB, while 19 (51.35%) of them are common in the 2 strains, 7 of which are potentially related to antibiotic resistance and highly conserved (~98-100% match ID) amongst the three strains of *E. rhusiopathiae* and only modestly homologous to other gastrointestinal tract-inhabiting Firmicutes (~40% match ID), e.g. *Clostridium* spp., *Enterococcus* spp. Molecular identification of these genomic elements and potential virulence factors offer insights into testing prospective vaccine targets besides the widely used SpaA antigens in swine erysipelas and development of more effective treatment or prevention in control of the disease.

## Competing interests

The authors declare that they have no competing interests.

## Authors’ contributions

AHYK prepared the genomic DNA for next-generation sequencing (NGS), post-NGS sequence alignment, genome assembly and annotation and comparative genomic analyses, and the drafting of the manuscript. YL and PJ isolated the bacteria from field sample and participated in design of study and coordination. JJ helped in writing in-house perl script and subsequent bioinformatics analyses. FCL participated in the design of study and coordination. All authors read and approved the final manuscript.

## Supplementary Material

Additional file 1 **Local Chinese swine erysipelas outbreak reports.** Three articles (titles shaded in yellow) on local swine erysipelas outbreaks published in Chinese scientific journals. Click here for file

Additional file 2 **NCBI nr database annotation for *E. rhusiopathiae *** **strain.** The full list of orfs in *E. rhusiopathiae* strain SY1027 annotated against NCBI non-redundant database. Click here for file

Additional file 3 **COG-annotated genes in *E. rhusiopathiae *** **strain SY1027 genome.** Tabulated summary of orfs in *E. rhusiopathiae* strain SY1027 annotated against COG database under various COG classes. Click here for file

Additional file 4 **COG functional annotation for *E. rhusiopathiae *** **strain SY1027 genome.** The full list of orfs in *E. rhusiopathiae* strain SY1027 annotated against COG database. Click here for file

Additional file 5 **COG class annotation distribution between *E. rhusiopathiae *** **strains SY1027 and Fujisawa genomes.** A bar chart comparing the percentage of each single-letter COG class found between the 2 complete *E. rhusiopathiae* genomes. Click here for file

Additional file 6 **Comparison of COG-annotated genes between *E. rhusiopathiae *** **strains SY1027 and Fujisawa.** Tabulated comparison between COG-annotated genes under each single-letter COG class from the 2 complete *E. rhusiopathiae* genomes. Click here for file

Additional file 7 **VFDB-annotated genes between *E. rhusiopathiae *** **strain SY1027 genome.** The list of VFDB-annotated genes in strain SY1027 genome, classified by COG classes. Genes shared between strains SY1027, Fujisawa and ATCC19414 were shaded in light gray while those shared between strains SY1027 and Fujisawa were highlighted in dark gray. Click here for file

Additional file 8 **Other potential virulence factors in *E. rhusiopathiae *** **strain SY1027 genome.** A panel of virulence factors mentioned in the literature was searched in strain SY1027 genome and listed here. ^orf00231, orf00462 and orf00466 are pseudogenes with frame-shift or point mutation(s). Click here for file

Additional file 9 **ARDB-annotated genes in *E. rhusiopathiae *** **strain SY1027 genome.** List of ARDB-annotated genes and their putative antibiotic resistance. Click here for file

Additional file 10 **ACLAME database-annotated genes in *E. rhusiopathiae *** **strain SY1027 genome.** Full list of orf annotated via ACLAME database in strain SY1027 genome. Click here for file

Additional file 11 **KEGG database-annotated genes in *E. rhusiopathiae *** **strain SY1027 genome.** Full list of KEGG database-annotated genes in strain SY1027 genome. Click here for file
