# Supplementary material for: Complete genome assembly and characterization of an outbreak strain of the causative agent of swine erysipelas – Erysipelothrix rhusiopathiae SY1027
Source: BMC Microbiol. 2014 Jul 2;14:176. doi: 10.1186/1471-2180-14-176 (PMC4105556; doi:10.1186/1471-2180-14-176)
Supplement: Additional file 1 — Local Chinese swine erysipelas outbreak reports. Three articles (titles shaded in yellow) on local swine erysipelas outbreaks published in Chinese scientific journals. [file 1471-2180-14-176-S1.pdf]

一起猪附红细胞体病诊治病例

欧朝顺　　付晓梅
（四川省西昌市畜牧局，四川　西昌　615000）

中图分类号：S858.28　　文献标志码：B　　文章编号：1008-0414(2013)11-0079-01

猪附红细胞体病是由附红细胞体寄生于猪的红细胞表面或游离于血浆、组织液及脑脊液中引起的一种人畜共患病，猪发病时，皮肤发红，故又称“猪红皮病”。以急性发病、黄疸贫血和发热为特征。

1　发病情况

起初有2~3头仔猪出现不愿活动，好睡，皮肤表面大面积发红现象，继而猪群中其它猪只也出现类似现象，畜主用抗生素和抗菌素治疗，效果不好，待出现病猪死亡现象后，畜主向兽医站报告。

2　临床症状

病猪精神不振，咳嗽、流鼻涕，体温40.5~42℃，呼吸困难，尿液淡黄、淡红或呈红褐色，皮肤发红，耳内侧、背侧、颈背部、腹侧部皮肤出现暗红色出血点，可视黏膜苍白、轻度黄疸，血液稀薄、色淡，濒死期四肢呈游泳状划动，呼吸困难，衰竭死亡。

3　病理变化

心包积液，血液稀薄；黏膜、浆膜、腹腔内的脂肪、肝脏等呈不同程度的黄染；全身淋巴结肿大；腹水增多，肺脏水肿，肝脾肿大，胆囊肿大，胆汁充盈，肾脏有出血点。

4　实验室检查

4.1　相差显微镜观察法

从病猪的耳尖采血，用玻璃棒蘸取血液一小滴，滴于载玻片的中央，盖上盖玻片，制成血液压片(也可制成悬滴标本)，置相差显微镜下进行观察。在许多红细胞上都发现有猪附红细胞体。

4.2　血片染色镜检

取死猪血液于载玻片上推片，以姬姆萨染色镜检，可见红细胞边缘不整齐，呈菜花状、星状，红细胞表面有许多圆形、椭圆形紫红虫体，轻轻旋动显微镜微调，可见一轮轮淡蓝色宝石（红细胞）中嵌着一颗颗发光的珍珠（附红细胞体），以瑞氏染色，可见虫体呈紫蓝色，个别为黄色，为阳性。

5　治疗

血虫净(或三氮脒、贝尼尔)5~10 mg/(kg体重)，用生理盐水稀释成5%溶液，分点肌肉注射，1次/d，连用3 d。辅以对症处置，3 d后症状消失。

6　讨论

6.1　由于猪附红细胞体病常伴有其他继发感染，因此对其治疗必须附以其他对症治疗才有较好的疗效。

6.2　猪附红细胞体病的发热、贫血、黄疸等症状具有一定的诊断意义，其他临床症状，如食欲减退、呼吸急迫、心悸亢进等非特征性症状在本病的诊断上意义不大。

6.3　注意与蓝耳病、猪气喘病、弓形体病、猪肺疫的鉴别诊断。

收稿日期：2013-09-18

及耳朵上，大牲畜和羊、狗、猫均可在颈静脉沟上1/3与中1/3交界处。此处肌肉较厚，静脉明显易见，便于操作。猪的静脉注射部位多在耳朵上。狗除在颈静脉外，也可在腕关节上的内侧静脉、跗关节外侧静脉、后肢内侧静脉等进行注射。

3.3　静脉注射操作方法

注射部位剪毛消毒后，以左手指压在注射部位近心端静脉上，静脉血管即可膨起，右手持相应粗细的针头，以15~45°角刺入静脉管内，见到回血后将针头顺血流方向推进1 cm，再徐徐注入药液。注药完毕后，左手持酒精棉球压紧针孔片刻以防血液外溢，最后涂擦碘酊消毒。如果静脉注射的药液是具有强烈刺激性的药液，如氯化钙、高渗葡萄糖盐水等，应防止药液渗漏于静脉血管之外。

3.4　静脉注射的特点

静脉注射时药液很快便由毛细血管进入组织，迅速出现药效，常用于急救及大量药液的输入，刺激性较大的药液和高渗溶液也可静注，因血流能迅速稀释药液，减少刺激性，但不可漏出血管外。混悬液和油溶液均不宜静注，宜使机体产生不良反应。

一例猪丹毒病的诊疗

唐　羽
（广西壮族自治区贺州市八步区里松镇水产畜牧兽医站，广西　贺州　542800）

中图分类号：S858.28　　文献标志码：B　　文章编号：1008-0414(2013)11-0079-02

猪丹毒是由红斑丹毒杆菌引起的一种急性、高热性的人畜共患传染病。该病多发于夏季高温季节，主要危害架子猪、肥猪和母猪，临床表现可分为“急性败血型”“亚急性皮肤疹块型”及“慢性心内膜炎和关节炎型”，常给猪场造成巨大损失。今年7月初，该镇一农户（存栏生猪150头），突然出现生猪群采食量下降，部分高烧不退、呼吸困难；陆续表现出急性败血症，心肺衰竭死亡；发病急，死亡快。发病第三天笔者应邀出诊。据农户反映，2 d内已发生猪急性死亡3头，群采食量整体下降至正常的2/3，不吃料的生猪近10头。发病生猪曾肌注磺胺六甲和安乃近，效果不佳，通过临床观察发现，发病生猪高烧41.5 ℃左右，部分背部，下颌部及面部皮肤出现典型不规则的“猪丹毒疹块”。食欲减退或完全停食，没有精神、寒战、病初粪便干燥后下痢、卧地不起，也有部分猪病后不久，在耳后、颈部、胸、腹侧等部位，（下转73页）

© 1994-2014 China Academic Journal Electronic Publishing House. All rights reserved.　http://www.cnki.net

浅谈猪胃肠臌气原因及防治

高新瑞[1]　汪秀菊[2]
（1.江苏省东海种猪场,江苏　东海　222300;2.江苏省东海畜禽改良站）

中图分类号:S858.28　文献标志码:B　文章编号:1008-0414(2013)11-0073-01

近几年，规模的养猪场发展较迅速,而发生猪胃肠鼓气比较多,本文就对猪胃肠臌气的原因及防治措施进行简单的分析。

1　微生物感染

1.1　大肠杆菌感染

幼龄猪在出生后3周以内极易感染大肠杆菌,其感染无季节限制。黄痢常波及一窝以内90%以上的仔猪发病,有的达100%,病死率很高;发生白痢时窝发病率可达30%~80%。病原性大肠杆菌具有多种毒力因子，使猪只发生腹泻和脱水，由于肠道的消化功能丧失，营养物质在肠道中发酵产生气体,引起肠道的臌气。

防治：仔猪一旦发生臌气常无好的治疗方法,故死亡率极高,运用敏感抗生素如头孢噻呋可抑制或杀灭病原,但对臌气却无作用,所以更重要的是预防。

1.2　产气荚膜梭菌感染

出生12 h至7日龄的仔猪易感染,更多见于3日龄，窝感染率为9%~l00%,病死率为5%~59%,平均死亡率为26%。 大部分猪会发生血痢,腹部皮肤变黑,精神衰弱,不愿走动。小肠严重出血,肠壁出现气肿,在其他肠管也会出现血样液体。

防治:一些药物如泰乐菌素、替硝唑对本菌有体外抑制作用，但实践中运用效果极差,故死亡率极高。母猪产前注射C型魏氏梭菌疫苗,以及仔猪初生后即灌服或注射敏感药物，配合改善环境和管理。

1.3　传染性胃肠炎病毒感染

传染性胃肠炎一般发生于冬、春季,可通过猪的接触传播,多呈地方性和周期性流行性。 病毒感染小肠上皮细胞后,使其迅速脱落,难以分解乳糖和其他必要的养分，由于渗透压的作用,从而引起水分的停留,甚至从身体组织中吸收体液,产生腹泻和失水。同时营养物质在肠道中发酵，产生大量气体,造成胃肠臌气。解剖尸体脱水明显,胃内充满凝乳块,肠管扩张呈半透明状,肠系膜充血。类似病变也可见于流行性腹泻。

防治：已发生臌气的临床病例无特效药物，对腹泻病例运用支持疗法和控制继发感染效果很明显。一方面补充体液和防止酸中毒，应用口服补液盐进行喂服。另一方面使用敏感抗生素防制继发感染,可选择头孢噻呋、小诺霉素等。也有干扰素对本病有效的报道。

1.4　感染性便秘

某些传染病、寄生虫病及其多病原混合感染例如猪瘟、蓝耳病、链球菌病、猪副嗜血杆菌病、慢性肠结核病、肠道蠕虫病等,能引起严重便秘,从而导致臌气，此外常伴发其它症状及病变。缓解便秘即能缓解臌气,在所谓无名高热的病例中,一些猪场进行通便、散结,缓解臌气,从而提高了治愈率。

防治:要制定科学的免疫程序,通过全方位的生物安全措施，来预防此类传染病的发生。

2　饲养管理不当

2.1　饲喂过饱

不定时的饲喂、饥饱不均等应激因素是导致过食的主要因素。病猪腹胀如鼓,胃壁扩张,造成消化障碍。通常在猪采食后15~20 min表现症状,病猪呼吸加快,眼球周边充血,严重的四肢不灵活,口鼻流黏液泡沫,肛门突出至死亡。

防治:可让猪停食1 d,少量饮水,同时可选择肥皂水或者生清油深部灌肠,并进行腹部按摩。促进胃的运动机能以及排空。对病情严重的猪可以注射比赛可林 (氨甲酰胆碱注射液),0.2 mL/kg,2次/d。

2.2　肠便秘

饲喂劣质饲料；饲料中混有多量泥沙;饮水不足,缺乏适当运动;断乳仔猪突然变换饲喂纯米糠而同时缺乏青绿饲料;伴有消化不良时的异嗜癖;去势引起肠粘连，甚至母猪去势时误将肠壁缝合在腹膜上等都可导致原发性肠便秘。便秘导致的胃肠臌气表现为食欲减退或废绝,饮欲增加,腹围增大,喜躺卧,有时呻吟,呈现腹痛,病初

收稿日期:2013-09-13

(上接 79 页)

皮肤上出现各种形状红斑，逐渐变为暗紫色,用手按压退色,停止按压时则又恢复,严重的脉搏纤细增快,呼吸困难,很快死亡。

剖检猪尸体表有形状各异和大小不同的暗红色疹斑。肠、胃都有炎症;脾肿大、充血、樱桃红色;肾肿大、暗红色,表面云雾状,皮质部内有暗红色小点;全身淋巴结肿大、紫红色;心包内有积水,心内膜点状出血。

综上症状判断,确定为猪丹毒病。

1　治疗方法

1.1　病猪用青霉素2万U/(kg体重)肌肉注射,2次/d。链霉素50 mg/(kg体重)肌肉注射,2次/d。

1.2　全场生猪同时结合用恩诺沙星300~500 g/t拌料，可溶性粉的阿莫西林300~400 g/t饮水。

1.3　全场生猪用多维500 mg/L,维C200 g/t,饮水。

经过采取以上措施后,3 d后疫情得到了有效的控制，猪群恢复正常采食。

近年来，由于猪场的饲养管理水平不断提高,广谱抗生素的广泛应用,典型的猪丹毒病例在规模化猪场已很少见,使很多养猪户产生了麻痹思想,疏于防范,使得该病在猪场又有“重新抬头”的迹象,特别是在一些饲养条件较差、管理松散的猪场更易发生。所以,养猪户还得提高专业知识,以免误诊,造成不必要的损失。

收稿日期:2013-09-29

© 1994-2014 China Academic Journal Electronic Publishing House. All rights reserved.　http://www.cnki.net

5.1 治疗

肌注氟苯尼考每千克体重 15~20 mg，每隔 48 h 一次，连用 2 次，收到了很好的治疗效果。

5.2 善后处理

保持鸡舍的通风和清洁卫生，供给鸡只营养丰富的饲料，精心饲养。保持鸡舍内合适的温湿度、饲养密度和光照。定期严格对鸡舍内外、饮水、饲料及用具等的消毒。及时清除粪便，勤换垫料，防止细菌滋生。尽量减少各种应激因素，同时做好球虫病等疾病的防治工作。

(收稿日期：2013-01-07)

仔猪流行性腹泻病的治疗

罗益民，薛太平，郭旭

(泰兴市畜牧兽医中心，江苏 泰兴 225400)

1 细菌性疾病引起的拉稀

大肠杆菌引起的仔猪黄痢常发生于 1 周龄以内，以 1~3 日龄者居多。仔猪白痢多发于生后 10~30 日龄的猪，以 10~20 日龄居多。治疗的药物有土碱粉、庆大+复 B 水，乳酶生、食母生等。

沙门细菌引起的拉稀，治疗药物土霉素、磺胺嘧啶 、阿莫西林、阿米卡星粉针、氨苄青等药物。

2 病毒性疾病引起的拉稀

目前尚无特效药物治疗，主要从抗病毒、防止细菌继发感染，提高机体抵抗力三方面着手。抗病毒药物有黄芪多糖、干扰素、免疫球蛋白、清开灵注射液等。防止细菌继发感染的药物有阿米卡星粉针、氨苄青、头孢曲松钠。对症治疗以减轻脱水、防止酸中毒为目的常用的药物有口服补液盐饮水，以调节电解质平衡，用于饮水。长期拉稀的仔猪使用收敛止泻药如活性炭、夕碳银等。同时要加强护理，做好防寒保暖工作。

3 寄生虫性疾病引起的拉稀

对于抗生素治疗效果不理想的拉稀，及时采集拉稀猪的粪便进行检查，如发现虫卵或有虫体就能确诊。线虫用伊维菌素或者左旋咪唑、丙硫咪唑(抗蠕敏)，球虫用磺胺类药物治疗。

4 饲养管理不善引起的拉稀

冬季注意防寒保暖，饲养密度要适宜，配制日粮营养要全价，适口性较好，不使用霉变和劣质饲料；尽量减少猪群转栏和混群的次数，减少应激反应，夏天做好灭蚊、灭蝇、灭鼠等工作。

(收稿日期：2012-11-01)

一例急性型猪丹毒的防控

谢祥有，许文珍

(会昌县畜牧兽医局，江西 赣州 342600)

猪丹毒为传统细菌性疾病，可以使用抗生素进行治疗。所以在免疫程序的安排上，不被重视。现介绍一例猪丹毒的病例，其防控措施供同行们参考。

1 发病情况

养殖户反映，50~100 kg 猪突发高烧，41℃~42℃，食欲废绝，行走摇摆不稳，全身皮肤泛红，耳朵、屁股、后肢内侧皮肤呈不规则紫斑块，指压红色暂时消退，去指后恢复原状。呼吸急迫，尿黄赤，有的表现神经症状，病程短，往往 1 d 左右死亡。以零星散发为主，发病率不高(约 10%)，死亡率几乎 100%。主要使用针对附红体、弓形体、热射病的药物，几乎无效。

2 病理变化

急性死亡的病猪，可见鼻、耳、颈部、胸腹及四肢，呈现不规则淡紫色。解剖病死猪，血凝不良、心脏疲软，脾肿大呈樱红色、包膜紧张、质脆。肺的心叶部分充血，肝脏有肿大淤血、暗红色。胃底部黏膜及小肠红肿，大肠无明显病变，淋巴结肿大，切面多汁。

针对以上临床表现和解剖病变，结合养户的用药治疗史，作者们初步确定是急性型猪丹毒。

3 治疗

治疗原则是清热消暑、杀菌消炎，肌肉注射清开灵水针、VC 注射液、头孢噻呋，首次用量是理论用量的加倍，后用维持量。饮水添加电解多维、板青颗粒冲剂、黄芪多糖，以消除热应激，提升抵抗力。连用 3 d，取得较好的防控效果。

4 防控措施

4.1 加强饲养管理

创造良好环境，提高猪群整体抵抗能力。

4.2 预防接种

10 kg 以上的断奶仔猪即可接种，猪丹毒 GC42 或 G4T10 弱毒苗、皮注 1 mL，7 d 产生免疫力，免疫期 6 个月；也可口服 2 mL，9 d 产生免疫力，免疫期 6 个月，免疫接种期间禁用抗生素。

4.3 治疗

早发现、早确诊、早治疗。首选用药是青霉素，静

© 1994-2013 China Academic Journal Electronic Publishing House. All rights reserved. http://www.cnki.net

2013 年 1 月第 4 周（上表）、5 周（下表）江西省部分县（市、区）畜产品及饲料市场零售价格

元/kg、元/只

集市名称	仔猪	活猪	猪肉	鸡蛋	蛋雏鸡	肉雏鸡	去骨牛肉	带骨羊肉	进口鱼粉	肥猪饲料	肉鸡饲料	蛋鸡饲料
南昌莲塘	34	18.4	26	10.8			54		13.6	3.3	3.2	3.2
乐平市区	28	19	30	11.9			60	56	15	3.9	3.5	3
湘东新街	32	17.6	23	13		3	39	47	6.8	3.2	3.3	
武宁古艾	22	17.6	29	13			60	64	13	3.4	3.4	3.2
德安县城	24	17.2	28	10.4			80	58	13	2.7	2.8	2.8
渝水城区	35	17.2	26	12	3	2.3	66			3.5	3.4	3.15
贵溪叶家塘	34	18.4	26.2	11			68	54	9.6	3.62	2.7	2.8
龙南县城	30	17	26	13	1.8	1.8	70	68	10	3.4	3.8	3.3
兴国潋江	17.5	16.6	24	9.5	0.6	0.5	66	53	10.1	3.4	3.3	3.4
寻乌长宁	30	17.4	26	12.6		2.2	70	80	10.2	3.3	3	3
南康蓉江	33.33	17.8	26.6	11.9	3.5	3	77	50		3.72	3.98	4.26
泰和县城	32	16	26	12		2.4	68	66	11.8	3.4	3.68	
永新县城	30	17.4	26	12.6		1.6	70	58	13.8	3.38	3.76	3.56
袁州区箭道	36	17.1	24.6	10.2	3.65	3.4	56	55.5		3.27	3.13	3.13
上高敖阳	28	16.6	26	12.6			70			3.34	4.14	3.54
樟树市区	27.4	17.4	23.5	10.3			63		10.7	3.39	3.35	3.32
崇仁桥南	22	18.8	26	13	0.8	1.7	76			3.22	3.26	3.08
资溪县城	34	18.6	26	12.6			64	46	6.8	3	3.4	3.3
东乡孝冈	34	17	28	12			63		12	3.58	3.54	3.52
上饶旭日	20	18.8	28	7	4	3.5	53	52		2.6	3	3.2
玉山冰溪	19	18	26	10.5			64	49		3.69	3.74	3.74
鄱阳县城	18	18	29	11.6			58			3.7	3.6	3
南昌莲塘	34.4	18.6	26	10.8			55		13.8	3.3	3.2	3.2
乐平市区	30	17.5	30	11.9		3	60	56	15	3.9	3.5	3
湘东新街	32	17.2	23	13.5			39	47	6.8	3.3	3.2	
武宁古艾	22	17.6	29	13			61	64	13	3.4	3.4	3.2
德安县城	26	17.6	28	10.4		2.3	80	58	13	2.7	2.8	2.8
渝水城区	35	16	26	11.8	3		66			3.5	3.4	3.15
贵溪叶家塘	34	19	27	12		1.8	68	54	9.6	3.62	2.7	2.8
龙南县城	30	16.8	26	13	1.8	0.5	70	68	10	3.4	3.8	3.3
兴国潋江	17.6	16.8	24	9.6	0.6	2.2	66	54	10.1	3.5	3.3	3.4
寻乌长宁	31	17.8	26	12.8		3	70	80	10.2	3.3	3	3
南康蓉江	40	16.8	25.6	11.9	3.5	2.4	78	50		3.72	3.98	4.26
泰和县城	32	16.8	26	12		1.6	70	66	11.8	3.4	3.68	
永新县城	31	17.2	26	12.6		3.5	70	58	13.8	3.38	3.76	3.56
袁州区箭道	36.2	17.2	24.8	10.3	3.7		56.2	56		3.28	3.14	3.14
上高敖阳	28	15.8	24	12.8			70			3.34	4.14	3.54
樟树市区	27.4	17.4	23.5	10.5		2	63		10.8	3.39	3.35	3.32
崇仁桥南	24	18.2	26	13	0.8		76			3.22	3.26	3.08
资溪县城	34	18.6	28	12.6			64	46	6.8	3	3.4	3.3
东乡孝冈	36	16.6	28	12		3.5	63		12	3.55	3.52	3.52
上饶旭日	20	19	28.5	7	4		53	52		2.6	3.1	3.2
玉山冰溪	18	17.6	26	10.5			62	51		3.69	3.74	3.74
鄱阳县城	18	18	29	11.6			58			3.8	3.6	3

（江西省畜牧兽医局提供）

注，每千克体重 1 万 u，同时按常规剂量肌肉注射，2~3 次/d，也可选用头孢噻呋等敏感药物。抗猪丹毒血清也可治疗本病。

5 小结

近几年来，受蓝耳病的影响，不少兽医人员对高烧不退，全身发红的猪病，一旦治疗效果不理想，发病势头不能及时遏止，即以“高热病”来解释。这样做，既敷衍了养户，也影响了自己的诊断结果。正确的诊断结论，应建立在望闻问切、饲养管理调查、气候环境变化的基础上。根据笔者的临床观察，像丹毒这类传统性细菌疾病有增多的迹象，而且临床表现形式与传统教科书所描述的症状有很大的出入，不容易及时被确诊。在此建议广大养猪业者在做好病毒性疾病防控的同时，也不可忽视对猪丹毒等传统性疾病的重视，应做到统筹安排、科学防控。

（收稿日期：2012-12-26）

© 1994-2013 China Academic Journal Electronic Publishing House. All rights reserved. http://www.cnki.net

"猪丹毒的现状、发病原因及应对措施"专栏二：

意想不到的猪丹毒复苏原因

张 蓉 译自《養豚界》2012 年 8 月第 28 ～ 33 页
潘雪男 校

很早以前猪丹毒就已是一种会严重影响养猪生产的疾病，即使近些年也有该病爆发的报道。但未能将其作为猪丹毒而采取错误应对措施的案例也不少。本文将总结性介绍 3 家养猪场的发病案例（表 1）。

表 1 猪场信息

所在地	东北地区
猪场数	3 家
规模	一贯经营 800 头、一贯经营 150 头、一贯经营 500 头
猪舍地面构造	800 头规模的猪场：肥育猪舍采用木屑和全部棚条式地面<br>150 头规模的猪场：肥育舍地面铺设木屑<br>500 头规模的猪场：肥育舍全部采用板条地面

中图分类号：S852 文献标识码：C 文章编号：1001-0769(2013)02-0009-6

1 序言

酷热的夏季大家是如何度过的呢？笔者十分敬佩在这炎热和艰难的养猪生产经营中，努力工作的养猪生产者和奋力生长的猪猪们，他们各自不断努力，力求在身体和精神两个方面都能健康生长。

于是，本公司就猪场在此项工作方式上出现的各种状况进行了电话咨询和面谈咨询。其中既有进行定期访问的猪场，也有初次接受本公司拜访的猪场。在这些咨询中，发现最近有些疾病的发生有增多的趋势，这正是猪丹毒。在之前的章节中曾以其为标题进行了介绍，但在此想再次针对猪丹毒病例进行详细讨论。

2 猪丹毒的认识

众所周知，猪丹毒是一种很久以前就已存在的疾病。然而，现状是同一地区既有接种疫苗的猪场又存在没有接种疫苗的猪场，就全日本国而言未接种疫苗的猪场占较高的比例。

大多数的猪场都认为"我们场没问题的"、"目前为止没有发生过该病"、"接种疫苗了就不用担心"，没有必要特意来防治猪丹毒 = 感觉上认为猪丹毒是一种让人不用担心的疾病。

3 至今未查明的猪丹毒

在此先预习了解一下猪丹毒的基本信息。

致病菌：红斑丹毒丝菌(*Erysipelothrix rhusiopathiae*)、革兰士阳性小杆菌

临床类型：败血症型、荨麻疹型、心内膜炎型、关节炎型 4 种

症状：败血症型会出现高烧发热、呼吸短促、呈现黑紫色发绀而死亡。

荨麻疹型会出现发热、食欲不良、全身出现淡红色丘疹（皮肤较嫩的地方也会出现丘疹）。

心内膜炎型可在心脏瓣膜的根部长出花椰菜型

发病率越来越大的猪丹毒，因是一种重要的人畜共患病，因此必须做好防止侵入的对策和接种灭活疫苗的预防措施。

每个猪场都切实采取相应的预防措施，对预防猪丹毒的发生非常重要。通过做好地区的猪丹毒发病情况的通告，并采取彻底的预防对策，可以预期今后猪丹毒的发生会得到减少。

原题名：豚丹毒とは農場にもたらす被害と対策（日文）

原作者：志贺 明（志贺猪诊所）

© 1994-2013 China Academic Journal Electronic Publishing House. All rights reserved. http://www.cnki.net

国外畜牧学—猪与禽
Animal Science Abroad-Pigs and Poultry
www.pig-poultry.cn www.shzyq.com

的肉芽（肿瘤），常见于发育迟缓的猪场和屠宰场中的猪。

关节炎型是继急性型后出现的，治愈完后或跟其无关的疾病引发的。红斑丹毒丝菌通过血液的流动，到达关节部位并定植，随后引发炎症而导致发病，轻度和严重的发病症状并不相同。

4 发病有增加趋势的猪丹毒

关节炎型猪丹毒在很多猪场中正悄悄地蔓延。但是，最近发病率正在增多的还是败血症型和荨麻疹型猪丹毒。

4.1 受感染较多的猪场的特征

笔者认为想考察一下实际情况中受感染较多的猪场的特征。

案例①：弱毒活疫苗接种量较少。

案例②：对仔猪接种弱毒活疫苗的时期过早，或过晚。

案例③：在接种活疫苗期间和前后服用了抗生素。

案例④：小母猪、经产母猪和公猪未接种疫苗。

案例⑤：仔猪未接种疫苗。

案例⑥：疫苗接种计划（次数、时间）不恰当。

案例⑦：接种灭活疫苗的时期较早。

案例⑧：灭活疫苗不受母源抗体的影响。

案例⑨：仅仅将弱毒活疫苗变更为灭活疫苗就认为可抑制发病。

案例⑩：对接种后用剩的弱毒活疫苗未进行适当的处理（丢弃在猪场内和猪舍中）。

案例⑾：没有采取必要的淘汰措施，长时间饲养发育不良的猪（仔猪和隔离猪）。

案例⑿：粪便尿液处理不当（粪坑斜道的刮粪次数、刮板的运作次数、堆肥设备的处理能力和地理条件等）。

案例⒀：死猪处理不当（未及时发现和处理、和堆肥场的关系等）。

虽然还存在除上述情况以外的原因和发病机制，但有必要注意可能会有意想不到的会导致疾病发生的因素存在。以上就是实地拜访的 3 家猪场情况。

5 被感染情况

以下商讨的是 3 家猪场和肥育猪舍中的急性发病案例。虽然无明显的腹泻和咳嗽症状，却出现了因精神快速衰弱而导致突然死亡的情况。发病年龄多在肥育期后期，集中在 120 ～ 180 日之间（图 1、2、3、4、5、6、7、8)。

猪场①：

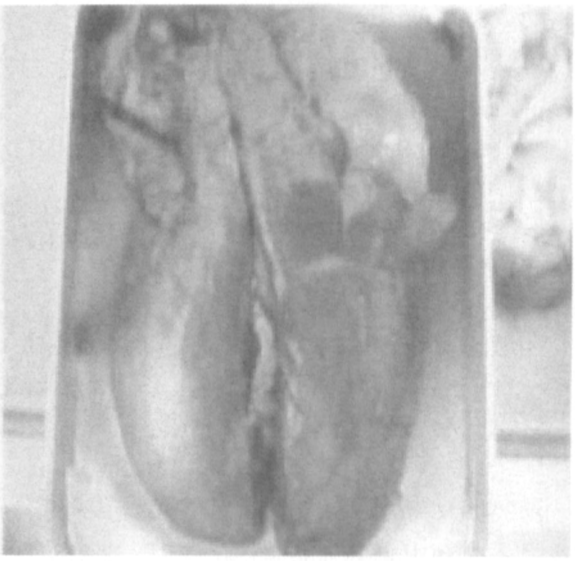

肺

猪场②

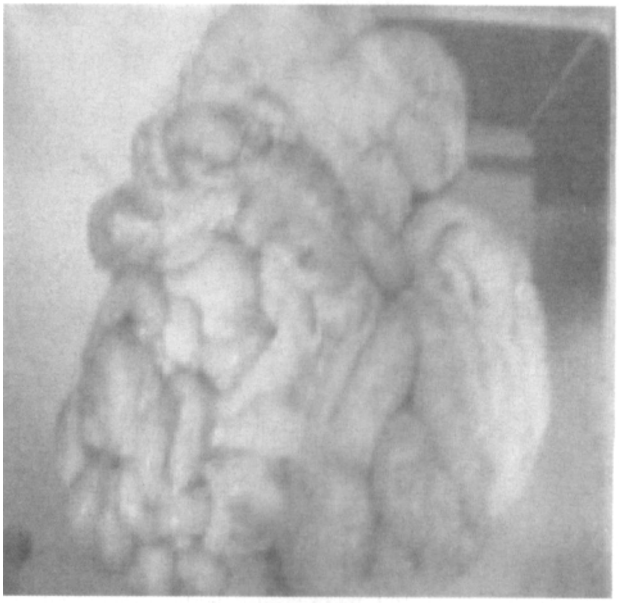

肠

根据发病症状，猪场多怀疑是猪胸肺膜炎(APP)，于是在可发挥功效的休药期内投服药物。但

© 1994-2013 China Academic Journal Electronic Publishing House. All rights reserved. http://www.cnki.net

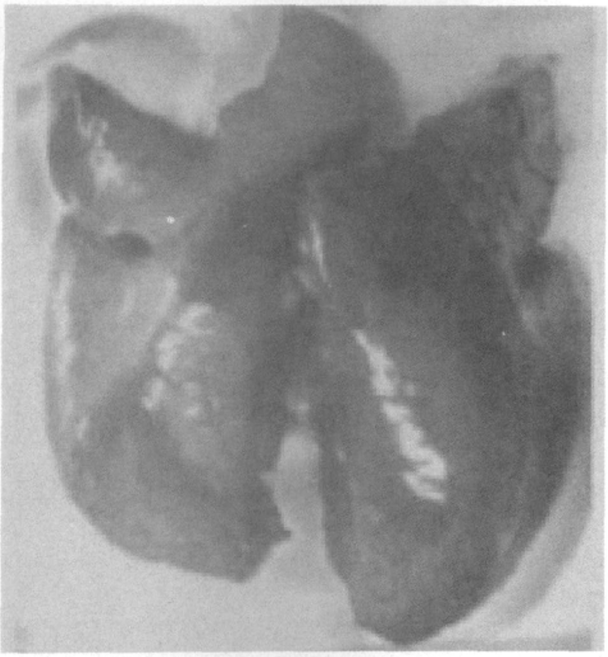

肺

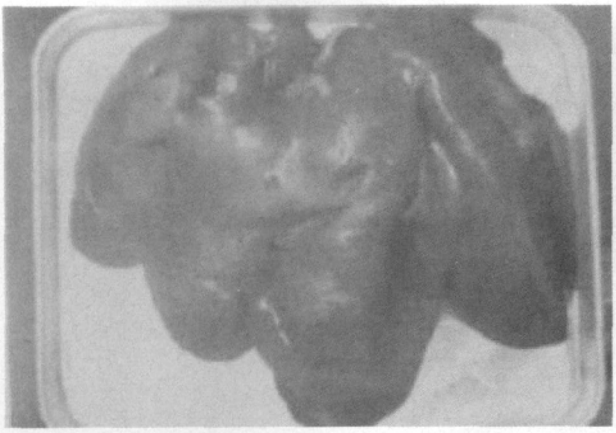

肝脏

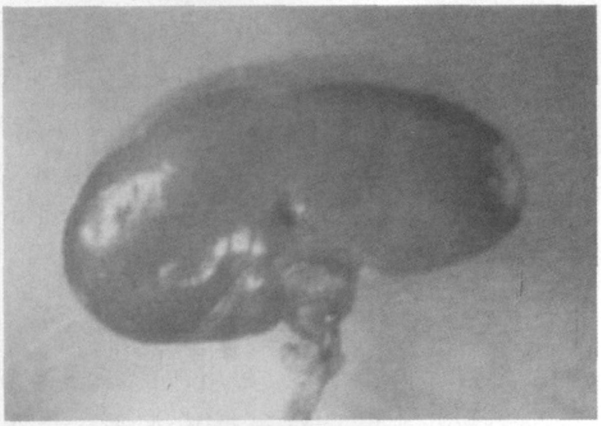

肾脏

是症状并没有减轻，发病率反而增加。

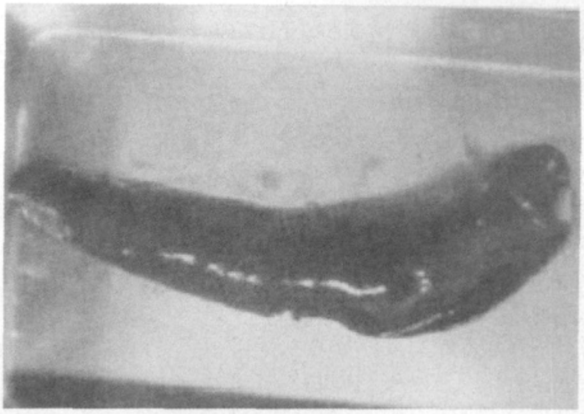

脾脏

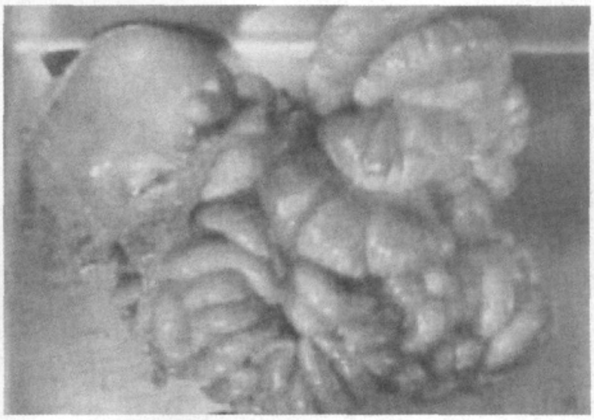

肠道

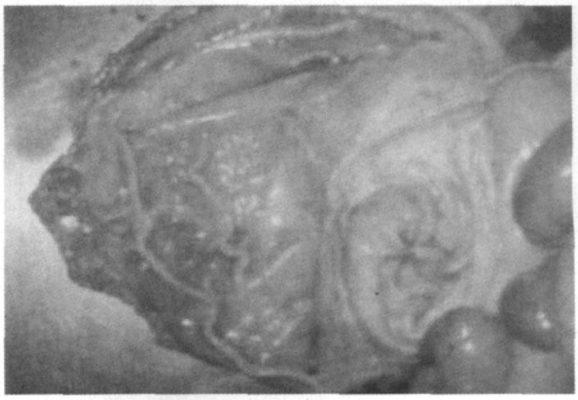

胃粘膜

笔者开始怀疑此病真的是由 APP 引发的吗？因此，对死亡猪的特征和使用的药物、疫苗接种计划等进行检查。结果显示，3 个案例中的死猪都出现了明显的发绀症状，它们周围到处可见猪只出现高烧发热、精力衰弱等症状。并且，这 3 家猪场都使用了对治疗 APP 有效的药物 (表 2、3)。

© 1994-2013 China Academic Journal Electronic Publishing House. All rights reserved. http://www.cnki.net

表 2　药物感受性表(猪场①)

抗生性		药物效果(1~3)	
名称	略称	猪丹毒	梭状芽孢杆菌
苄基青霉素	PCG	3	3
氨苄青霉素	ABPC	3	3
阿莫西林	AMPC	3	3
传染试剂	ASPC	3	3
甲亚胺青霉素	MPC	0	0
头孢噻呋	CTF	3	3
非处方药	OTC	2	1
多西环素	DOXY	3	3
四氧化炭	CTC	3	3
链霉素	SM	1	0
卡那霉素	KM	0	0
庆大霉素	GM	0	0
胸苷酸合成酶	TS	2	3
四甲基硅烷	TMS	2	3
奥索利酸	OA	2	3
恩诺沙星	ERFX	3	3
达氟沙星	DNFX	3	3
奥比沙星	OBFX	3	3
氟苯尼考	FF	3	3
血清总蛋白	TP	3	3
ST 合剂	ST	0	0
rinkomaishin	LCM	3	1
氯化物	CL	1	0
二环霉素	BCM	0	0
硫姆林	TML	1	0

注：效果：3：+++　2：++　1：+　0：—(+++表示效果非常好、—表示效果很差)。
检查结果是对实验室内效果的评价，与实际临床效果可能会不同。

表 3　药物感受性表(猪场②)

抗生性		药物效果(1~3)		
名称	略称	猪丹毒	大肠杆菌 K88	梭状芽孢杆菌
苄基青霉素	PCG	3	0	3
氨苄青霉素	ABPC	3	3	3
阿莫西林	AMPC	3	3	3
传染试剂	ASPC	3	3	3
甲亚胺青霉素	MPC	0	3	0
头孢噻呋	CTF	3	3	3
非处方药	OTC	2	0	2
多西环素	DOXY	3	2	3
四氧化炭	CTC	3	3	3
链霉素	SM	1	2	0
卡那霉素	KM	0	2	0
胸苷酸合成酶	TS	2	0	1
四甲基硅烷	TMS	1	0	0
奥索利酸	OA	2	3	2
恩诺沙星	ERFX	3	3	3
达氟沙星	DNFX	3	3	3
奥比沙星	OBFX	3	3	2
氟苯尼考	FF	3	3	3
血清总蛋白	TP	3	2	2
ST 合剂	ST	3	0	0
rinkomaishin	LCM	0	0	0
氯化物	CL	0	1	0
二环霉素	BCM	3	1	0
硫姆林	TML	0	0	1

注：效果：3：+++　2：++　1：+　0：—(+++表示效果非常好、—表示效果很差)。
检查结果是对实验室内效果的评价，与实际临床效果可能会不同。

表 4　疾病鉴定结果(猪场①)

检查项目	检查方法	结果
猪胸膜肺炎杆菌(A．pp)	从肺部分离病菌	阴性(-)
副猪嗜血杆菌(H．ps)	从肺部分离病菌	阴性(-)
多杀性巴氏杆菌(P.m)	从肺部分离病菌	阴性(-)
沙门氏菌	从肺部分离病菌	阴性(-)
链球菌(Str.suis)	从心血管分离病菌	阴性(-)
猪丹毒(E.rhusiopathiae)	从心血管分离病菌	阳性(+)
猪繁殖与呼吸综合征(PRRS)	在肺部和淋巴处使用 PCR	阴性(-)
猪圆环病毒 2 型(PCV2)	在淋巴处使用 PCR	阴性(-)
病原大肠杆菌(vero 毒素和线毛型)	从肠道内物质分离病菌	阴性(-)
沙门氏菌	从肠道内物质分离病菌	阴性(-)
梭状芽孢杆菌	从肠道内物质分离病菌	7.1×10^{7} cfu/g
寄生虫	用游浮法肠镜检查肠道内物质	阴性(-)
寄生虫	用姬姆萨染色法肠镜检查肠道内物质	阴性(-)
聚苯醚(PPE) 胞内劳森氏菌猪增生性肠炎	在肠道处使用 PCR	阴性(-)

6 检查结果

6.1 检查结果①

通过对死猪内脏进行病原菌检查，分离出了猪丹毒菌；病菌感染到血液和肝脏等脏器，引发了败血症 (表 4、5)。

6.2 检查结果②

利用活的猪丹毒菌检测抗体水平，结果发现发病前和发病后的免疫水平出现了显

© 1994-2013 China Academic Journal Electronic Publishing House. All rights reserved.　http://www.cnki.net

表 5 疾病鉴定结果(猪场②)

检查项目	检查方法	结果
聚磷酸铵(*A.pp*)	从肺部分离病菌	阴性(-)
副猪嗜血杆菌(*H.ps*)	从肺部分离病菌	阴性(-)
多杀性巴氏杆菌(*P.m*)	从肺部分离病菌	阴性(-)
沙门氏菌	从肺部分离病菌	阴性(-)
链球菌(*Str.suis*)	从心血管分离病菌	阴性(-)
猪丹毒(*E.rhusiopathiae*)	从心血管分离病菌	阳性(+)
猪繁殖与呼吸综合征(PRRS)	在肺部和淋巴处使用 PCR	阴性(-)
猪圆环病毒 2 型(PCV2)	在淋巴处使用 PCR	阴性(-)
病原大肠杆菌(vero 毒素和线毛型)	从肠道内物质分离病菌	vero 毒素非产生大肠杆菌 K88
沙门氏菌	从肠道内物质分离病菌	阴性(-)
梭状芽孢杆菌	从肠道内物质分离病菌	1.1×10^5 cfu/g
寄生虫	用游浮法肠镜检查肠道内物质	阴性(-)
寄生虫	用姬姆萨染色法肠镜检查肠道内物质	阴性(-)
聚苯醚(PPE) 胞内劳森氏菌猪增生性肠炎	在肠道处使用 PCR	阴性(-)

著的变化(表 6、7、8)。

7 调整应对措施

7.1 措施①：调整免疫接种计划

虽然 2 个案例猪场都接种了活疫苗，但因为接种时间(一个案例在 50 日龄前接种，另一个在 120 日龄接种)和药物使用方法(2 件案例都在接种疫苗前后给猪服用了药物)的不正确，所以改为接种灭活疫苗。根据猪场的现状，这两个猪场的疫苗接种时间选定在 6 ～ 10 周龄左右和 9 ～ 13 周龄左右。

在另一个案例中，猪场原本接种弱毒活疫苗，因发病改为接种灭活疫苗，由于没有出现预想的效果，因此对接种时间再次进行了考察。因为 4 ～ 7 周龄左右接种疫苗后 120 日龄后还是会发病，所以将疫苗接种时间改为 6 ～ 10 周龄左右。

7.2 措施②：再次检查药物使用计划

发病猪群服用喹诺酮系列的新药，在出栏之前猪则服用青霉素系列药物。为了确保无猪丹毒菌的残留，无论采用哪一种方法都应注意药物的使用剂量。并且，为防止疾病的爆发，重新考虑仔猪群、种猪群的投药方案，建议采用基于四环素和盘尼西林系列药物的预防方案。

7.3 措施③：死猪的处理和猪舍的消毒

死猪鼻涕、唾液、血液、粪便、尿和内脏器官等携带的大量猪丹毒病菌暴露在猪场内。在进行将死猪从猪舍中搬出、处置和解剖等作业时，都要采用适当的保护措施。为了避免猪丹毒菌的循环污染，猪舍和过道都应进行彻底的消毒。特别要注意的是，不要共用猪场内使用过的长靴、铲子和重型机械(重型机械等也要彻底清洁和消毒)。

7.4 措施④：改变认识

向接种弱毒活疫苗的猪场解释：活疫苗与抗生素间的关系、活疫苗接种后的正确处理和灭活疫苗的特性等。向使用灭活疫苗的猪场解释：仅仅用灭活疫苗替换弱毒活疫苗并非那么重要。另外，向 3 家猪场同时解释有关粪尿处理、消毒、工具使用方法和死猪处置等的方法，注意提高与猪丹毒发病相关联的管理意识。

3 家猪场在执行各项措施后，猪群的症状都有所改善，至今为止再也没有猪丹毒死灰复燃的迹象。笔者接到这样的报告后也终于放心了。

8 总结

近年来，猪圆环病毒 2 型 (PCV2 型) 的再次爆发和猪繁殖与呼吸综合症 (PRRS) 新毒株的侵入等成为人们关注的主要话题，而很早以前就已存在的疾病往往会被人们所忽视。事实上，无论毒力多强的病毒侵入，其所造成损害的程度一定会对细菌性疾病的存在产生巨大的影响。

© 1994-2013 China Academic Journal Electronic Publishing House. All rights reserved. http://www.cnki.net

表 6　抗体检查(猪场①)

No.	检查物名称	SE-WP	
		发病前	发病后
1		32	8
2		8	16
3	育成猪	8	8
4		4	8
5		4	16
6		8	8
7		8	6
8		4	32
9		8	8
10	分娩猪舍	32	32
11		8	8
12		8	32
13		16	8
14		16	16
15		16	16
16		16	8
17		32	16
18	定位栏 配种后 30 d	16	16
19		16	16
20		32	128
21		8	16
22		16	16
23	定位栏 配种后 30 d	8	32
24		16	32
25		32	32
26		<4	<4
27		<4	<4
28	哺乳猪	<4	4
29		<4	<4
30		8	<4
31		<4	<4
32		4	<4
33	60 日龄猪	<4	4
34		<4	<4
35		<4	4
36		4	4
37		<4	<4
38	90 日龄猪	4	<4
39		4	<4
40		<4	<4
41		4	4
42		4	<4
43	120 日龄猪	4	8
44		4	4
45		4	4
46		4	4
47		8	4
48	150 日龄猪	8	8
49		8	8
50		8	8

表 7　抗体检查(猪场②)

No.	检查物名称	SE-WP	
		发病前	发病后
1		<4	4
2		<4	<4
3	100 日龄	4	8
4		<4	4
5		<4	4
6		4	32
7		8	16
8	140 日龄	4	8
9		8	256≤
10		4	16
11		8	8
12		8	4
13	170 日龄	8	4
14		8	128
15		8	256≤

表 8　抗体检查(猪场③)

No.	检查物名称	SE-WP	
		发病前	发病后
1		8	8
2		8	8
3		4	8
4		4	8
5	出栏前	8	16
6		4	16
7		8	256≤
8		8	16
9		8	8
10		8	8

今后，加强能够预防外部新型强毒病毒和细菌等侵入的生物安全是非常重要的。另一方面，控制很早就已存在的疾病发生对稳定猪场的生产经营也尤为重要。

现实的问题是，同一地区相同条件下的同一种疾病即使有关联，然而其在不同猪场中所造成的影响却存在差异。虽然不同猪场所隐藏的疾病和病毒的毒力各不相同，但实际中除此之外的案例也时有发生。

虽然还不明白为何会出现这样的现象，但似乎能够从不同猪场的经营意识（管理方法、管理意识、防疫规定、目标等）中找到答案。世事就是如此，虽然显而易见的答案就摆在面前，但却兜兜转转地将问题越弄越复杂，大概因为缺少简单、灵活的思考方式和想象力吧。

原题名：豚丹毒が再燃する意外な理由（日文）

原作者：山本贤休兽医师、菊池雄一兽医师（食用环境卫生研究所）

© 1994-2013 China Academic Journal Electronic Publishing House. All rights reserved.　http://www.cnki.net
