# Supplementary material for: Complete genome assembly and characterization of an outbreak strain of the causative agent of swine erysipelas – Erysipelothrix rhusiopathiae SY1027
Source: BMC Microbiol. 2014 Jul 2;14:176. doi: 10.1186/1471-2180-14-176 (PMC4105556; doi:10.1186/1471-2180-14-176)
Supplement: Additional file 3 — COG-annotated genes in E. rhusiopathiae strain SY1027 genome. Tabulated summary of orfs in E. rhusiopathiae strain SY1027 annotated against COG database under various COG classes. [file 1471-2180-14-176-S3.pdf]

COG class	Number of COG class	Percentage of COG class	Class Function	COG class	Number of COG class	Percentage of COG class	COG class	Number of COG class	Percentage of COG class
[R]	292	12.08	General function prediction only	[TK]	22	0.91	[LU]	2	0.08
[J]	276	11.41	Translation, ribosomal structure and biogenesis	[KR]	16	0.66	[CP]	2	0.08
[G]	218	9.02	Carbohydrate transport and metabolism	[KG]	14	0.58	[FGR]	2	0.08
[L]	206	8.52	Replication, recombination and repair	[KT]	12	0.50	[NT]	2	0.08
[S]	176	7.28	Function unknown	[GEPR]	12	0.50	[EM]	2	0.08
[K]	150	6.20	Transcription	[MG]	10	0.41	[IQR]	2	0.08
[P]	132	5.46	Inorganic ion transport and metabolism	[Q]	8	0.33	[NU]	2	0.08
[E]	128	5.29	Amino acid transport and metabolism	[LKJ]	8	0.33	[IQ]	2	0.08
[V]	112	4.63	Defense mechanisms	[EP]	6	0.25	[ET]	2	0.08
[M]	104	4.30	Cell wall/membrane/envelope biogenesis	[QR]	6	0.25	[KL]	2	0.08
[C]	98	4.05	Energy production and conversion	[HI]	6	0.25	[EH]	2	0.08
[F]	92	3.80	Nucleotide transport and metabolism	[CHR]	6	0.25	[CO]	2	0.08
[O]	74	3.06	Posttranslational modification, protein turnover, chaperones	[LK]	4	0.17	[LR]	2	0.08
[T]	46	1.90	Signal transduction mechanisms	[OC]	4	0.17	[EF]	2	0.08
[H]	44	1.82	Coenzyme transport and	[GT]	4	0.17	[TQ]	2	0.08
[I]	36	1.49	Lipid transport and metabolism	[IR]	4	0.17	[FE]	2	0.08
[D]	30	1.24	Cell cycle control, cell division, chromosome partitioning	[MU]	2	0.08	[CR]	2	0.08
[U]	24	0.99	Intracellular trafficking, secretion, and vesicular transport	[MI]	2	0.08			
