# Supplementary figures and images for: Complete genome assembly and characterization of an outbreak strain of the causative agent of swine erysipelas – Erysipelothrix rhusiopathiae SY1027
Source: BMC Microbiol. 2014 Jul 2;14:176. doi: 10.1186/1471-2180-14-176 (PMC4105556; doi:10.1186/1471-2180-14-176)

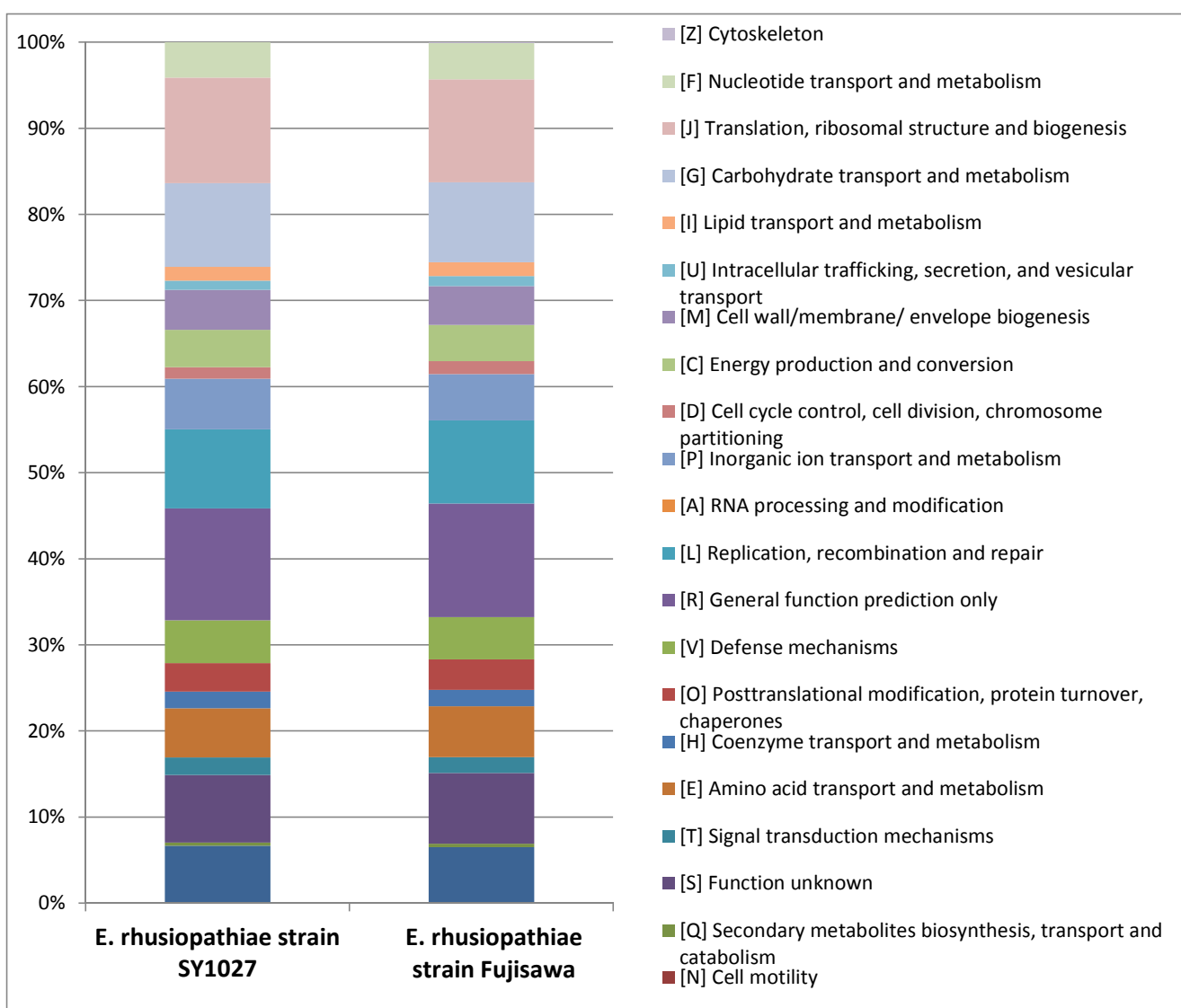

Supplement: Additional file 5 — COG class annotation distribution between E. rhusiopathiae strains SY1027 and Fujisawa genomes. A bar chart comparing the percentage of each single-letter COG class found between the 2 complete E. rhusiopathiae genomes. [file 1471-2180-14-176-S5.pdf]
