# Supplementary material for: Complete genome assembly and characterization of an outbreak strain of the causative agent of swine erysipelas – Erysipelothrix rhusiopathiae SY1027
Source: BMC Microbiol. 2014 Jul 2;14:176. doi: 10.1186/1471-2180-14-176 (PMC4105556; doi:10.1186/1471-2180-14-176)
Supplement: Additional file 6 — Comparison of COG-annotated genes between E. rhusiopathiae strains SY1027 and Fujisawa. Tabulated comparison between COG-annotated genes under each single-letter COG class from the 2 complete E. rhusiopathiae genomes. [file 1471-2180-14-176-S6.pdf]

[COG class] function description	*Erysipelothrix rhusiopathiae* strain SY1027		*Erysipelothrix rhusiopathiae* strain Fujisawa	
	no. of ORF	%	no. of ORF	%
[K] Transcription	75	6.203	68	6.055
[N] Cell motility	0	0.000	0	0.000
[Q] Secondary metabolites biosynthesis, transport and catabolism	4	0.331	4	0.356
[S] Function unknown	88	7.279	86	7.658
[T] Signal transduction mechanisms	23	1.902	19	1.692
[E] Amino acid transport and metabolism	64	5.294	62	5.521
[H] Coenzyme transport and metabolism	22	1.820	20	1.781
[O] Posttranslational modification, protein turnover, chaperones	37	3.060	37	3.295
[V] Defense mechanisms	56	4.632	51	4.541
[R] General function prediction only	146	12.076	138	12.289
[L] Replication, recombination and repair	103	8.519	101	8.994
[A] RNA processing and modification	0	0.000	0	0.000
[P] Inorganic ion transport and metabolism	66	5.459	56	4.987
[D] Cell cycle control, cell division, chromosome partitioning	15	1.241	16	1.425
[C] Energy production and conversion	49	4.053	44	3.918
[M] Cell wall/membrane/ envelope biogenesis	52	4.301	47	4.185
[U] Intracellular trafficking, secretion, and vesicular transport	12	0.993	12	1.069
[I] Lipid transport and metabolism	18	1.489	17	1.514
[G] Carbohydrate transport and metabolism	109	9.016	97	8.638
[J] Translation, ribosomal structure and biogenesis	138	11.414	125	11.131
[F] Nucleotide transport and metabolism	46	3.805	44	3.918
[Z] Cytoskeleton	0	0.000	1	0.089
