# Supplementary material for: Complete genome assembly and characterization of an outbreak strain of the causative agent of swine erysipelas – Erysipelothrix rhusiopathiae SY1027
Source: BMC Microbiol. 2014 Jul 2;14:176. doi: 10.1186/1471-2180-14-176 (PMC4105556; doi:10.1186/1471-2180-14-176)
Supplement: Additional file 8 — Other potential virulence factors in E. rhusiopathiae strain SY1027 genome. A panel of virulence factors mentioned in the literature was searched in strain SY1027 genome and listed here. ^orf00231, orf00462 and orf00466 are pseudogenes with frame-shift or point mutation(s). [file 1471-2180-14-176-S8.pdf]

Locus Tag	Size (a.a.)	Alignment length	% ID	GenBank accession no.	Gene description
**Surface proteins**					
contig00001_orf00166	1559	1556	97.94	ZP_08081965	Family 85 glycosyl hydrolase
contig00001_orf00230^	1005	1003	99.60	YP_004560766	Surface protein A (rspA)
contig00001_orf00232^	971	945	99.89	YP_004560766	Surface protein A (rspA)
contig00001_orf00233	790	790	100.00	YP_004560767	Surface protein B (rspB)
contig00001_orf00308	510	510	99.80	YP_004560825	Leucine-rich repeat protein
contig00001_orf00352	1557	1533	100.00	YP_004560861	hyaluronidase (hyl B)
contig00001_orf00355	607	586	100.00	YP_004560864	Choline-binding protein
contig00001_orf00364	588	584	98.47	YP_004560873	Dipeptidase
contig00001_orf00807	754	721	99.86	YP_004561235	5'-nucleotidase
contig00001_orf00890	850	838	99.88	YP_004561305	hyaluronidase (hyl C)
contig00001_orf00949	102	98	91.84	YP_004561353	Hypothetical protein
contig00001_orf01192	1308	1308	100.00	YP_004561530	collagen-binding protein
contig00001_orf01214	726	726	100.00	YP_004561548	LPXTG-motif cell wall anchor domain-containing protein
contig00001_orf01488	1062	1059	100.00	YP_004561780	Surface protein C (rspC)
contig00001_orf01594	1773	1773	99.94	YP_004560175	collagen-binding protein
contig00001_orf01621	627	626	99.68	YP_004560194	spaA
contig00001_orf01689	1034	1034	100.00	YP_004560250	hyaluronidase (hyl A)
contig00001_orf01700	1174	1174	99.91	YP_004560261	Peptidase M14
contig00001_orf01704	1487	1487	99.93	YP_004560265	Pectin lyase fold-containing protein
contig00001_orf01831	632	632	100.00	YP_004560378	LPXTG-motif cell wall anchor domain-containing protein
contig00001_orf01852	1198	1198	99.92	YP_004560398	neuraminidase
**Antioxidant proteins**					
contig00001_orf00708	203	203	100.00	YP_004561161	sodA superoxide dismutase
contig00001_orf01039	292	292	100.00	YP_004561405	trxB.1 thioredoxin-disulfide reductase
contig00001_orf01077	118	118	100.00	YP_004561439	ahpD alkylhydroperoxidase-like
contig00001_orf01270	102	102	100.00	YP_004561594	trxA.2 thioredoxin
contig00001_orf01317	311	311	100.00	YP_004561635	trxB.2 thioredoxin-disulfide reductase
contig00001_orf01701	162	162	100.00	YP_004560262	tpx thiol peroxidase
contig00001_orf01717	221	221	100.00	YP_004560275	ahpC alkyl-hydroperoxide reductase
contig00001_orf01922	69	69	100.00	YP_004560455	nrdH glutaredoxin-like protein NrdH
contig00001_orf01943	103	103	100.00	YP_004560474	trxA.1 thioredoxin
**Phospholipase**					
contig00001_orf01588	302	302	100.00	YP_004560172	patatin-like phospholipase
contig00001_orf01607	197	197	100.00	YP_004560183	phospholipase/carboxylesterase family protein
contig00001_orf01686	275	275	100.00	YP_004560248	pldB lysophospholipase
contig00001_orf01892	508	508	100.00	YP_004560432	cls cardiolipin synthetase
contig00001_orf01893	284	284	100.00	YP_004560433	patatin-like phospholipase
contig00001_orf01910	200	200	100.00	YP_004560446	phospholipase/carboxylesterase
**Hemolysins**					
contig00001_orf00060	424	424	100.00	YP_004560565	hemolysin-like protein
contig00001_orf00209	219	219	100.00	YP_004560747	hemolysin III
**Other extracellular proteins/enzymes**					
contig00001_orf00348	407	407	100.00	YP_004560857	nanH.2 neuraminidase
contig00001_orf00669	193	193	100.00	YP_004561130	fibronectin-binding protein
contig00001_orf01091	292	292	100.00	YP_004561450	ABC transporter metal-binding
contig00001_orf01232	493	493	88.03	YP_004561561	putative biofilm-associated surface protein
**Capsular polysaccharide biosynthesis**					
contig00001_orf00461^	442	442	100.00	YP_004560951	exopolysaccharide biosynthesis polyprenyl
contig00001_orf00463^	502	502	99.80	YP_004560952	putative O-antigen polymerase
contig00001_orf00464	401	401	100.00	YP_004560953	glycosyltransferase
contig00001_orf00465^	337	337	100.00	YP_004560954	UDP-glucose 4-epimerase
contig00001_orf00467^	293	293	100.00	YP_004560955	NAD-dependent
contig00001_orf00468	374	374	100.00	YP_004560956	UDP-N-acetylglucosamine 2-epimerase
contig00001_orf00469	391	390	100.00	YP_004560957	glycosyltransferase
