# Supplementary material for: Complete genome assembly and characterization of an outbreak strain of the causative agent of swine erysipelas – Erysipelothrix rhusiopathiae SY1027
Source: BMC Microbiol. 2014 Jul 2;14:176. doi: 10.1186/1471-2180-14-176 (PMC4105556; doi:10.1186/1471-2180-14-176)
Supplement: Additional file 9 — ARDB-annotated genes in E. rhusiopathiae strain SY1027 genome. List of ARDB-annotated genes and their putative antibiotic resistance. [file 1471-2180-14-176-S9.pdf]

Species and contigs in E. rhusiopathiae SY1027	ARDB best hit					Ortholog in strain Fujisawa
	Match ID (%)	Accession no.	Gene	Potential resistance to	Description on ARDB	
contig00001_orf00313	40.10	NP_836538	macb	macrolide	macrolide transporter/permease	YP_004560830
contig00001_orf00863	41.84	YP_001176128	macb	macrolide	macrolide transporter/permease	YP_004561283
contig00001_orf01097	40.36	YP_001334578	macb	macrolide	macrolide transporter/permease	YP_004561455
contig00001_orf01260	43.06	NP_836538	macb	macrolide	macrolide transporter/permease	YP_004561586
contig00001_orf01760	42.06	ABA71727	vanrg	vancomycin	VanG type vancomycin resistance operon genes, which can synthesize peptidoglycan with modified C-terminal D-Ala-D-Ala to D-alanine--D-serine.	YP_004560311
contig00001_orf01866	45.18	AAL27445	vanre	vancomycin	VanE type vancomycin resistance operon genes, which can synthesize peptidoglycan with modified C-terminal D-Ala-D-Ala to D-alanine--D-serine.	YP_004560408
contig00001_orf01870	46.86	AAY52009	vanra	teicoplanin	Unknown	YP_004560411
